# Tuning CRISPR-Cas9 Gene Drives in *Saccharomyces cerevisiae*

**DOI:** 10.1534/g3.117.300557

**Published:** 2018-01-18

**Authors:** Emily Roggenkamp, Rachael M. Giersch, Madison N. Schrock, Emily Turnquist, Megan Halloran, Gregory C. Finnigan

**Affiliations:** * Department of Biochemistry and Molecular Biophysics, Kansas State University, Manhattan, Kansas 66506; † Department of Biology, Kansas State University, Manhattan, Kansas 66506

**Keywords:** CRISPR, Cas9, budding yeast, gene drive, sgRNA, regulating gene drives, biotechnology

## Abstract

Control of biological populations is an ongoing challenge in many fields, including agriculture, biodiversity, ecological preservation, pest control, and the spread of disease. In some cases, such as insects that harbor human pathogens (*e.g.*, malaria), elimination or reduction of a small number of species would have a dramatic impact across the globe. Given the recent discovery and development of the CRISPR-Cas9 gene editing technology, a unique arrangement of this system, a nuclease-based “gene drive,” allows for the super-Mendelian spread and forced propagation of a genetic element through a population. Recent studies have demonstrated the ability of a gene drive to rapidly spread within and nearly eliminate insect populations in a laboratory setting. While there are still ongoing technical challenges to design of a more optimal gene drive to be used in wild populations, there are still serious ecological and ethical concerns surrounding the nature of this powerful biological agent. Here, we use budding yeast as a safe and fully contained model system to explore mechanisms that might allow for programmed regulation of gene drive activity. We describe four conserved features of all CRISPR-based drives and demonstrate the ability of each drive component—Cas9 protein level, sgRNA identity, Cas9 nucleocytoplasmic shuttling, and novel Cas9-Cas9 tandem fusions—to modulate drive activity within a population.

The issue of biological control over the population, spread, and nature of a given species has been a monumental challenge that has major implications for agriculture, human health, and the environment. Many metazoans, including insects, are carriers for human pathogens such as malaria. Plant, fungal, and insect species are also pests that can affect planting, pollination, harvesting, transportation, or storage of agricultural products. Finally, the introduction of invasive species (accidental or malicious) into a nonnative environment can disrupt ecological food chains, impact biodiversity, or inflict damage on human infrastructure. Current measures to combat these widespread issues include a full range of solutions, such as chemical treatment (and genetically modified crops), physical barriers, and natural predators, and each mechanism has its benefits and limitations (Stenberg *et al.* 2015; Kergunteuil *et al.* 2016; Khan *et al.* 2016; Peterson *et al.* 2016; Jonsson *et al.* 2017). However, in situations that seem to call for the near eradication of an entire single species type (*e.g.*, insect genus *Anopheles* as a vector for the malaria-causing alveolate parasites from genus *Plasmodium*, or the tsetse fly from genus *Glossina* for sleeping sickness caused by protozoa genus *Trypanosoma*), no solution has been developed with the ability to selectively control populations.

The discovery of the CRISPR (Clustered Regularly Interspaced Short Palindromic Repeats) gene editing system has revolutionized the entire fields of molecular and cellular biology and genetics, providing a new level of control over the genomes of all living species. Briefly, the Cas9 nuclease from *Streptococcus pyogenes* was identified as a critical part of a bacterial immune system used to identify, bind, and destroy invading bacteriophage DNA sequences (Sorek *et al.* 2013; Mohanraju *et al.* 2016; Koonin *et al.* 2017). The CRISPR system was recognized for its biotechnological application as a programmable genome editor: given a particular DNA sequence, a double-stranded break (DSB) could be introduced at a precise chromosomal location. The Cas9 protein is first primed by a single guide RNA (sgRNA) fragment containing a CRISPR RNA (crRNA) sequence (typically 20 bp) that contains the corresponding bases to the intended genomic target fused to the tracer RNA (tracrRNA) sequence, a species-specific structural nucleic fragment that complexes with the Cas9 nuclease (Jinek *et al.* 2012). The bound Cas9/sgRNA complex then scans DNA for a feature only present within the target nucleotide sequence, called the protospacer adjacent motif (PAM). For *S. pyogenes* Cas9, the sequence 5′-NGG-3′ found at the 3′ end of the intended target sequence allows the nuclease to create a DNA DSB either *in vitro* or *in vivo* (Jinek *et al.* 2012, 2014; Nishimasu *et al.* 2014; Jiang *et al.* 2016). Following cleavage, eukaryotic cells have evolved several DNA repair pathways that activate to fix the Cas9-induced break including nonhomologous end joining (NHEJ) or, in the presence of exogenous donor DNA, homology directed repair (HDR). The combination of Cas9-targeted genomic breaks coupled with native repair systems allows for precise editing (deletion, disruption, replacement) of nearly any genomic sequence. The power and potential of the CRISPR-Cas9 editing system cannot be overstated: a myriad of research, biotechnology, agricultural, and medical industries in both the public and private sectors have begun applying this genomic editing system to many current problems in biology and human health (Doudna and Charpentier 2014; Sternberg and Doudna 2015; Barrangou and Doudna 2016; Estrela and Cate 2016; Wright *et al.* 2016; Baltes *et al.* 2017).

One unique arrangement of Cas9 has garnered a lot of interest in recent years, the nuclease based “gene drive.” Briefly, both the Cas9 gene and the expression cassette for the sgRNA are integrated at a particular genomic locus (usually disrupting and replacing a given gene). The nuclease function of Cas9 is unique within the context of a gene drive because the intended target for the guide RNA is the WT copy of the gene that has been disrupted by placement of the drive-expression cassette (DiCarlo *et al.* 2015). Upon induction of a DSB within a diploid cell, the homologous chromosome pair serves as the DNA “donor” and, via HDR, will copy the entire gene drive (Cas9 and its sgRNA) to the second chromosome, replacing and deleting the WT copy of the target gene in the process. This results in the transformation of every heterozygous pairing to be converted to a homozygous diploid state; “forced” propagation of a desired loss of function allele through a population is super-Mendelian in nature as it defies the basic mechanisms of heredity. In this way, the introduction of a single affected individual harboring one copy of the gene drive can rapidly sweep through a population in only a few generations. The identity of the targeted gene(s) may differ (early renditions have used a biased male/female sex ratio to reduce population sizes), but the goal is the same: the preprogrammed, automated destruction of the entire population of (only) the given species. This potent biological agent would, theoretically, have the potential to rapidly introduce a given allele of choice to modify millions (or billions) of individuals for any intended purpose with no external input. To date, only a few laboratories (Gantz *et al.* 2015; Hammond *et al.* 2016; Champer *et al.* 2017; Drury *et al.* 2017) have demonstrated use of a CRISPR gene drive in insects and a single study piloted the technology in yeast (DiCarlo *et al.* 2015). There are still many technical hurdles to overcome in the design and application of gene drives into wild populations, such as evolved resistance to the action of the drive, genetic diversity within populations, and containment/reversal of drive systems. Furthermore, there are serious ecological and ethical considerations regarding the issue of actual use of gene drives to modify (or eradicate) native populations, regardless of the intended purpose (Akbari *et al.* 2015; Esvelt and Gemmell 2017). Therefore, we have focused our efforts on an important, and currently overlooked aspect of gene drives for future implementation and application in the wild: a programmable and tunable drive system that would result in a full spectrum of drive efficiencies (from 0 up to 99%) within a population.

In this study, we have developed an artificial genetic system in budding yeast to (i) provide maximum containment and biosafety, (ii) rapidly explore and test many aspects of the CRISPR editing system using the available molecular tools within *Saccharomyces cerevisiae*, and (iii) identify and characterize sources within the gene drive system for future control and regulation. We describe the study of conserved gene drive components regardless of the organism of choice: the Cas9 nuclease, its subcellular localization, and the sgRNA. We demonstrate that the following independent mechanisms can all modulate drive activity to varying degrees: (i) the level of Cas9 (using an inducible promoter), (ii) the sgRNA length and mismatch to the target sequence, (iii) nucleocytoplasmic shuttling of Cas9 using localization signals, and (iv) tandem fusions between Cas9(s) and/or a nuclease dead (dCas9) variant. Moreover, these alterations can be done in combination resulting in a wide spectrum of gene drive penetrance within a population from zero activity to maximal activity. These findings represent an important step in the future design, regulation, and possible application of gene drives in the wild.

## Materials and Methods

### Yeast strains and plasmids

*S. cerevisiae* strains used in this study are shown in Table 1. Standard molecular and cellular biology procedures were used to manipulate all plasmids and yeast strains (Sambrook and Russell 2001). The Cas9 gene was synthesized *de novo* with a yeast codon bias (Genscript, Piscataway, NJ). The second Cas9* gene used for the tandem fusions was also synthesized after manual manipulation of each codon to an alternate codon (primarily within the Wobble position). Enzymatically dead Cas9 (D10A H840A) was generated by a modified PCR mutagenesis protocol (Zheng *et al.* 2004) on the pUC57-based plasmid(s) harboring the Cas9 gene using a high-fidelity DNA polymerase (KOD Hot Start; EMD Millipore). The general strategy for integration of Cas9 (or the target gene cassette) into the yeast genome was as follows. First, a CEN-based (pRS316) plasmid was generated by *in vivo* plasmid assembly (Finnigan and Thorner 2015) including the *GAL1/10* promoter (814 bp), Cas9 open reading frame (ORF), a C-terminal NLS signal (SRADPKKKRKV), the *ADH1* terminator (238 bp) (Bennetzen and Hall 1982), and the MX-based kanamycin resistance cassette (Longtine *et al.* 1998). Second, the assembled Cas9 gene cassette was PCR amplified and a second round of *in vivo* ligation was performed using a second vector (pGF-IVL974) to insert 992 bp of *HIS3* 5′ UTR, 993 bp 3′ UTR, and two (u2) sites (Finnigan and Thorner 2016) upstream of the *GAL1/10* promoter and downstream of the MX terminator. Third, the entire ensemble was amplified in two fragments (generating 120 bp of overlapping sequence within the Cas9 ORF), treated with *Dpn*I enzyme, and transformed into BY4741 yeast using a modified lithium acetate protocol (Eckert-Boulet *et al.* 2012) for integration at the native *HIS3* locus (*his3*∆*1*). Colonies resistant to G418 sulfate (and lacking the selectable marker for the yeast vector used as a PCR template) were tested by diagnostic PCR and Sanger DNA sequencing (Genscript) to generate GFY-2383.

**Table 1:** Yeast strains used in this study

Strain	Genotype	Reference
BY4741	*MAT****a*** *his3*Δ*1 leu2*Δ*0 met15*Δ*0 ura3*Δ*0*	Brachmann *et al.* (1998)
BY4742	*MAT***⍺** *his3*Δ*1 leu2*Δ*0 lys2*Δ*0 ura3*Δ*0*	Brachmann *et al.* (1998)
GFY-2353*^a^*	BY4741; *his3*∆::*(u1)*::*prMX*::*Hyg^R^*::*MX(t)*::*(u1)*::*HIS3(t)*	This study
GFY-2588*^b^*	BY4741; *his3*∆::*(u1)*::*prMX*::*Kan^R^*::*MX(t)*::*(u1)*::*HIS3(t)*	This study
GFY-2383*^c^*	BY4741; *his3*∆::*(u2)*::*prGAL1/10*::*SpCas9*::*NLS*::*ADH1(t)*::*Kan^R^*::*(u2)*::*HIS3(t)*	This study
GFY-3206*^d^*	BY4742; *his3*∆::*(u1)*::*prCDC12*::*mCherry*::*NLS*::*SHS1(t)*::*prCCW12*::*SpHIS5*::*MX(t)*::*(u1)*::*HIS3(t)*	This study
GFY-3207	BY4742; *his3*∆::*(u1)*::*prCDC12*::*mCherry*::*SHS1(t)*::*prCCW12*::*SpHIS5*::*MX(t)*::*(u1)*::*HIS3(t)*	This study
GFY-2751*^e^*	BY4741; *his3*∆::*(u2)*::*prGAL*::*SpCas9*::*NLS*::*eGFP*::*ADH1(t)*::*Kan^R^*::*(u2)*::*HIS3(t)*	This study
GFY-2752	BY4741; *his3*∆::*(u2)*::*prGAL*::*SpCas9*::*NLS*::*eGFP*::*NLS*::*ADH1(t)*::*Kan^R^*::*(u2)*::*HIS3(t)*	This study
GFY-2753	BY4741; *his3*∆::*(u2)*::*prGAL*::*SpCas9*::*NLS*::*eGFP*::*NES*::*ADH1(t)*::*Kan^R^*::*(u2)*::*HIS3(t)*	This study
GFY-2754	BY4741; *his3*∆::*(u2)*::*prGAL*::*SpCas9*::*NLS*::*eGFP*::*NLS*::*NES*::*ADH1(t)*::*Kan^R^*::*(u2)*::*HIS3(t)*	This study
GFY-2755	BY4741; *his3*∆::*(u2)*::*prGAL*::*SpCas9*::*eGFP*::*ADH1(t)*::*Kan^R^*::*(u2)*::*HIS3(t)*	This study
GFY-2756	BY4741; *his3*∆::*(u2)*::*prGAL*::*SpCas9*::*eGFP*::*NLS*::*ADH1(t)*::*Kan^R^*::*(u2)*::*HIS3(t)*	This study
GFY-3101	BY4741; *his3*∆::*(u2)*::*prGAL*::*SpCas9*::*eGFP*::*NES*::*ADH1(t)*::*Kan^R^*::*(u2)*::*HIS3(t)*	This study
GFY-2758	BY4741; *his3*∆::*(u2)*::*prGAL*::*SpCas9*::*eGFP*::*NLS*::*NES*::*ADH1(t)*::*Kan^R^*::*(u2)*::*HIS3(t)*	This study
GFY-2759	BY4741; *his3*∆::*(u2)*::*prGAL*::*NLS*::*SpCas9*::*NLS*::*eGFP*::*ADH1(t)*::*Kan^R^*::*(u2)*::*HIS3(t)*	This study
GFY-2760	BY4741; *his3*∆::*(u2)*::*prGAL*::*NLS*::*SpCas9*::*NLS*::*eGFP*::*NLS*::*ADH1(t)*::*Kan^R^*::*(u2)*::*HIS3(t)*	This study
GFY-2761	BY4741; *his3*∆::*(u2)*::*prGAL*::*NLS*::*SpCas9*::*NLS*::*eGFP*::*NES*::*ADH1(t)*::*Kan^R^*::*(u2)*::*HIS3(t)*	This study
GFY-2762	BY4741; *his3*∆::*(u2)*::*prGAL*::*NLS*::*SpCas9*::*NLS*::*eGFP*::*NLS*::*NES*::*ADH1(t)*::*Kan^R^*::*(u2)*::*HIS3(t)*	This study
GFY-2763	BY4741; *his3*∆::*(u2)*::*prGAL*::*NLS*::*SpCas9*::*eGFP*::*ADH1(t)*::*Kan^R^*::*(u2)*::*HIS3(t)*	This study
GFY-2764	BY4741; *his3*∆::*(u2)*::*prGAL*::*NLS*::*SpCas9*::*eGFP*::*NLS*::*ADH1(t)*::*Kan^R^*::*(u2)*::*HIS3(t)*	This study
GFY-2765	BY4741; *his3*∆::*(u2)*::*prGAL*::*NLS*::*SpCas9*::*eGFP*::*NES*::*ADH1(t)*::*Kan^R^*::*(u2)*::*HIS3(t)*	This study
GFY-2766	BY4741; *his3*∆::*(u2)*::*prGAL*::*NLS*::*SpCas9*::*eGFP*::*NLS*::*NES*::*ADH1(t)*::*Kan^R^*::*(u2)*::*HIS3(t)*	This study
GFY-3250*^f^*	BY4741; *his3*∆::*(u2)*::*prGAL*::*SpCas9(D10A H840A)*::*NLS*::*ADH1(t)*::*Kan^R^*::*(u2)*::*HIS3(t)*	This study
GFY-3099*^g^*	BY4741; *his3*∆::*(u2)*::*prGAL*::*SpCas9(D10A H840A)*::*Link*::SpCas9*::*NLS*::*ADH1(t)*::*Kan^R^*::*(u2)*::*HIS3(t)*	This study
GFY-3100	BY4741; *his3*∆::*(u2)*::*prGAL*::*SpCas9*::*Link*::SpCas9(D10A H840A)*::*NLS*::*ADH1(t)*::*Kan^R^*::*(u2)*::*HIS3(t)*	This study
GFY-3336	BY4741; *his3*∆::*(u2)*::*prGAL*::*SpCas9*::*Link*::SpCas9*::*NLS:;ADH1(t)*::*Kan^R^*::*(u2)*::*HIS3(t)*	This study
GFY-3264*^h^*	BY4741; *his3*∆::*(u2)*::*prGAL*::*SpCas9*::*NLS*::*eGFP*::*ADH1(t)*::*Kan^R^*::*(u2)*::*HIS3(t) NUP188*::*mCherry*::*ADH1(t)*::*SpHIS5*	This study 18
GFY-3265	BY4741; *his3*∆::*(u2)*::*prGAL*::*SpCas9*::*NLS*::*eGFP*::*NLS*::*ADH1(t)*::*Kan^R^*::*(u2)*::*HIS3(t) NUP188*::*mCherry*::*ADH1(t)*::*SpHIS5*	This study 19
GFY-3266	BY4741; *his3*∆::*(u2)*::*prGAL*::*SpCas9*::*NLS*::*eGFP*::*NES*::*ADH1(t)*::*Kan^R^*::*(u2)*::*HIS3(t) NUP188*::*mCherry*::*ADH1(t)*::*SpHIS5*	This study 20
GFY-3267	BY4741; *his3*∆::*(u2)*::*prGAL*::*SpCas9*::*NLS*::*eGFP*::*NLS*::*NES*::*ADH1(t)*::*Kan^R^*::*(u2)*::*HIS3(t) NUP188*::*mCherry*::*ADH1(t)*::*SpHIS5*	This study 21
GFY-3270	BY4741; *his3*∆::*(u2)*::*prGAL*::*SpCas9*::*eGFP*::*NES*::*ADH1(t)*::*Kan^R^*::*(u2)*::*HIS3(t) NUP188*::*mCherry*::*ADH1(t)*::*SpHIS5*	This study 24
GFY-3271	BY4741; *his3*∆::*(u2)*::*prGAL*::*SpCas9*::*eGFP*::*NLS*::*NES*::*ADH1(t)*::*Kan^R^*::*(u2)*::*HIS3(t) NUP188*::*mCherry*::*ADH1(t)*::*SpHIS5*	This study 25
GFY-3273	BY4741; *his3*∆::*(u2)*::*prGAL*::*NLS*::*SpCas9*::*NLS*::*eGFP*::*NLS*::*ADH1(t)*::*Kan^R^*::*(u2)*::*HIS3(t) NUP188*::*mCherry*::*ADH1(t)*::*SpHIS5*	This study 27
GFY-3275	BY4741; *his3*∆::*(u2)*::*prGAL*::*NLS*::*SpCas9*::*NLS*::*eGFP*::*NLS*::*NES*::*ADH1(t)*::*Kan^R^*::*(u2)*::*HIS3(t) NUP188*::*mCherry*::*ADH1(t)*::*SpHIS5*	This study 29

a The “unique Cas9 target site” (u1) contains the sequence 5′ ATGA**CGGTGGACTTCGGCTACGTA**GGGCGATT 3′ where the 20 bp target site is in bold and the PAM sequence is underlined. This (u1) sequence was inserted directly flanking the Hyg^R^ MX-based cassette and integrated at the native *HIS3* locus in BY4741 WT yeast by amplifying the entire locus from pGF-IVL1143.

b The Hyg^R^ cassette was replaced with the Kan^R^ cassette. Strain GFY-2588 is otherwise isogenic to GFY-2353.

c The Cas9-expressing gene drive strain is flanked by (u2) sites at the *HIS3* locus of the sequence 5′ **GCTGTTCGTGTGCGCGTCCT**GGG 3′ where the 20 bp target site is in bold and the PAM sequence is underlined.

d The gene drive target locus contains 448 bp of 5′ UTR of the *CDC12* gene, 486 bp of 3′ UTR of the *SHS1* gene, and 992 bp of 5′ UTR of the *CCW12* gene. The *S. pombe HIS5* gene is the functional equivalent to *S. cerevisiae HIS3*.

e Strains GFY-2751 – GFY-2756, GFY-2758 – GFY-2766, and GFY-3101 were constructed by first generating plasmids containing the Cas9-expression cassettes from pGF-IVL1162 through pGF-IVL1177 flanked by (u2) sites and *HIS3* 5′ and 3′ UTR (plasmids pGF-IVL1318–pGF-IVL1333, respectively) using *in vivo* plasmid assembly. Next, the entire cassette was PCR amplified in two fragments using overlapping primers within the coding sequence of the Cas9 gene, transformed into BY4741 WT yeast, and integrated at the *HIS3* locus. Each strain was confirmed by DNA sequencing of PCR amplified fragments spanning the entire expression cassette and flanking UTR.

f The catalytic dead mutations (D10A and H840A) were mutagenized by a modified Quikchange protocol (Zheng *et al.* 2004) in the pUC57 vector prior to assembly by *in vivo* ligation in yeast. The dCas9 expression cassette was first assembled into pGF-IVL1180 followed by a second round of assembly to include flanking (u2) sites and *HIS3* 5′ and 3′ UTR. The entire cassette was PCR amplified and integrated at the *HIS3* locus.

g GFY-3099, GFY-3100, and GFY-3336 were constructed by the following methodology. First, two parental plasmids were constructed by *in vivo* assembly containing either *prGAL-SpCas9(D10A H840A)-SpeI-ADH1(t)-Kan^R^* or *prGAL-SpCas9-SpeI-ADH1(t)-Kan^R^* (pGF-IVL1312 and pGF-IVL1313, respectively). A 15-residue flexible linker sequence (GRRIPGLINGGSSGS) was also inserted in-frame at the C-terminus of Cas9. Second, a second SpCas9 gene (designated SpCas9*) was synthesized *de novo* with >90% of all codons changed to an alternate sequence (if possible), primarily within the Wobble position (to provide maximum mismatch between the two identical copies of SpCas9 and prevent homologous recombination between the tandem genes). Third, digestion with *SpeI* and a second round of *in vivo* ligation including the amplified SpCas9* (either a WT or catalytically dead mutant version) created a tandem fusion between dCas9-Cas9* (pGF-IVL1345) and Cas9-dCas9* (pGF-IVL1346B). Attempts to perform a third round of *in vivo* ligation (to include the flanking (u2) and *HIS3* UTR sequences) were unsuccessful. Therefore, the fourth step included direct integration at the *HIS3* locus with four overlapping PCR fragments (treated with *DpnI*) from pGF-IVL1396 and pGF-1345 (to construct GFY-3099) or pGF-IVL1192 and pGF-IVL1346B (to construct GFY-3100) in a single transformation event. For GFY-3336, similar PCR fragments were generated from the same set of parental vectors harboring WT Cas9 (native or Wobble variants). Confirmation of these strains included multiple diagnostic PCRs and DNA sequencing of the entire locus.

h Strains GFY-2751–GFY-2756, GFY-2758–GFY-2766, and GFY-3101 were transformed with an amplified PCR fragment of the C-terminus of *NUP188* fused to *mCherry-ADH1(t)-SpHIS5* from a chromosomal DNA preparation from GFY-1517.

DNA plasmids used in this study are shown in Table 2. *In vivo* plasmid assembly was used to construct all Cas9 and gene target vectors including those used for integration into the genome. Plasmids expressing the sgRNA cassette were created from previous vectors (DiCarlo *et al.* 2013; Finnigan and Thorner 2016). Briefly, the *SNR52* promoter (269 bp), crRNA sequence (16–22 bp), tracrRNA sequence (79 bp), and *SUP4* terminator (20 bp) were synthesized *de novo* (Genscript), subcloned into the pRS423, pRS425, or pRS426 (high-copy) plasmids (Christianson *et al.* 1992), and mutagenized by PCR to generate all sgRNA variants. All vectors were confirmed by Sanger DNA sequencing (Genscript).

**Table 2:** Plasmids used in this study

Plasmid	Description	Reference
pRS315	*CEN*, *LEU2*	Sikorski and Hieter (1989)
pRS316	*CEN*, *URA3*	Sikorski and Hieter (1989)
pRS425	*2*μ, *LEU2*	Christianson *et al.* (1992)
pRS426	*2*μ, *URA3*	Christianson *et al.* (1992)
pGF-IVL1116*^a^*	pRS316; *prGAL1/10*::*SpCas9*::*NLS*::*ADH1(t)*::*Kan^R^*	This study
pGF-IVL1342	pRS316; *prGAL1/10*::*SpCas9*::*NLS*::*ADH1(t)*::*Hyg^R^*	This study
pGF-IVL1119	pRS316; *prGAL1/10*::*SpCas9*::*eGFP*::*NLS*::*ADH1(t)*::*Kan^R^*	This study
pGF-IVL1180	pRS316; *prGAL1/10*::*SpCas9(D10A H840A)*::*NLS*::*ADH1(t)*::*Kan^R^*	This study
pGF-IVL1183	pRS316; *prGAL1/10*::*SpCas9(D10A H840A)*::*eGFP*::*NLS*::*ADH1(t)*::*Kan^R^*	This study
pGF-V809*^b^*	pRS425; *prSNR52*::*Sp-sgRNA(u2-20 WT)*::*SUP4(t)*	This study
pGF-V798*^b^*	pRS423; *prSNR52*::*Sp-sgRNA(u2-20 WT)*::*SUP4(t)*	Finnigan and Thorner (2016)
pGF-V1215*^c^*	pRS315; *prSNR52*::*Sp-sgRNA(u1-20WT)*::*SUP4(t)*	This study
pGF-V1216*^d^*	pRS425; *prSNR52*::*Sp-sgRNA(u1-16WT)*::*SUP4(t)*	This study
pGF-V1217	pRS425; *prSNR52*::*Sp-sgRNA(u1-17WT)*::*SUP4(t)*	This study
pGF-V1218	pRS425; *prSNR52*::*Sp-sgRNA(u1-18WT)*::*SUP4(t)*	This study
pGF-V1219	pRS425; *prSNR52*::*Sp-sgRNA(u1-19WT)*::*SUP4(t)*	This study
pGF-V1220	pRS425; *prSNR52*::*Sp-sgRNA(u1-20WT)*::*SUP4(t)*	This study
pGF-V1625	pRS426; *prSNR52*::*Sp-sgRNA(u1-20WT)*::*SUP4(t)*	This study
pGF-V1221	pRS425; *prSNR52*::*Sp-sgRNA(u1-21WT)*::*SUP4(t)*	This study
pGF-V1222	pRS425; *prSNR52*::*Sp-sgRNA(u1-22WT)*::*SUP4(t)*	This study
pGF-V1223*^e^*	pRS425; *prSNR52*::*Sp-sgRNA(u1-17-1mut)*::*SUP4(t)*	This study
pGF-V1224	pRS425; *prSNR52*::*Sp-sgRNA(u1-18-1mut)*::*SUP4(t)*	This study
pGF-V1225	pRS425; *prSNR52*::*Sp-sgRNA(u1-19-1mut)*::*SUP4(t) [5′ G→A]*	This study
pGF-V1797	pRS425; *prSNR52*::*Sp-sgRNA(u1-19-1mut)*::*SUP4(t) [5′ G→C]*	This study
pGF-V1799	pRS425; *prSNR52*::*Sp-sgRNA(u1-19-1mut)*::*SUP4(t) [5′ G→T]*	This study
pGF-V1226	pRS425; *prSNR52*::*Sp-sgRNA(u1-20-1mut)*::*SUP4(t)*	This study
pGF-V1227	pRS425; *prSNR52*::*Sp-sgRNA(u1-21-1mut)*::*SUP4(t)*	This study
pGF-V1228	pRS425; *prSNR52*::*Sp-sgRNA(u1-22-1mut)*::*SUP4(t)*	This study
pGF-V1229*^e^*	pRS425; *prSNR52*::*Sp-sgRNA(u1-17-2mut)*::*SUP4(t)*	This study
pGF-V1230	pRS425; *prSNR52*::*Sp-sgRNA(u1-18-2mut)*::*SUP4(t)*	This study
pGF-V1231	pRS425; *prSNR52*::*Sp-sgRNA(u1-19-2mut)*::*SUP4(t)*	This study
pGF-V1232	pRS425; *prSNR52*::*Sp-sgRNA(u1-20-2mut)*::*SUP4(t)*	This study
pGF-V1233	pRS425; *prSNR52*::*Sp-sgRNA(u1-21-2mut)*::*SUP4(t)*	This study
pGF-V1234	pRS425; *prSNR52*::*Sp-sgRNA(u1-22-2mut)*::*SUP4(t)*	This study
pGF-V1235*^e^*	pRS425; *prSNR52*::*Sp-sgRNA(u1-17-3mut)*::*SUP4(t)*	This study
pGF-V1236	pRS425; *prSNR52*::*Sp-sgRNA(u1-18-3mut)*::*SUP4(t)*	This study
pGF-V1237	pRS425; *prSNR52*::*Sp-sgRNA(u1-19-3mut)*::*SUP4(t)*	This study
pGF-V1238	pRS425; *prSNR52*::*Sp-sgRNA(u1-20-3mut)*::*SUP4(t)*	This study
pGF-V1239	pRS425; *prSNR52*::*Sp-sgRNA(u1-21-3mut)*::*SUP4(t)*	This study
pGF-V1240	pRS425; *prSNR52*::*Sp-sgRNA(u1-22-3mut)*::*SUP4(t)*	This study
pGF-V1241*^f^*	pRS425; *prSNR52*::*Sp-sgRNA(u1-17-1Del)*::*SUP4(t)*	This study
pGF-V1242	pRS425; *prSNR52*::*Sp-sgRNA(u1-18-1Del)*::*SUP4(t)*	This study
pGF-V1243	pRS425; *prSNR52*::*Sp-sgRNA(u1-19-1Del)*::*SUP4(t)*	This study
pGF-V1244	pRS425; *prSNR52*::*Sp-sgRNA(u1-20-1Del)*::*SUP4(t)*	This study
pGF-V1245	pRS425; *prSNR52*::*Sp-sgRNA(u1-21-1Del)*::*SUP4(t)*	This study
pGF-V1246	pRS425; *prSNR52*::*Sp-sgRNA(u1-22-1Del)*::*SUP4(t)*	This study
pGF-IVL1162*^g^*	pRS316; *prGAL*::*SpCas9*::*NLS*::*eGFP*::*ADH1(t)*::*Kan^R^*	This study
pGF-IVL1163	pRS316; *prGAL*::*SpCas9*::*NLS*::*eGFP*::*NLS*::*ADH1(t)*::*Kan^R^*	This study
pGF-IVL1164*^h^*	pRS316; *prGAL*::*SpCas9*::*NLS*::*eGFP*::*NES*::*ADH1(t)*::*Kan^R^*	This study
pGF-IVL1165*^i^*	pRS316; *prGAL*::*SpCas9*::*NLS*::*eGFP*::*NLS*::*NES*::*ADH1(t)*::*Kan^R^*	This study
pGF-IVL1166	pRS316; *prGAL*::*SpCas9*::*eGFP*::*ADH1(t)*::*Kan^R^*	This study
pGF-IVL1167	pRS316; *prGAL*::*SpCas9*::*eGFP*::*NLS*::*ADH1(t)*::*Kan^R^*	This study
pGF-IVL1168	pRS316; *prGAL*::*SpCas9*::*eGFP*::*NES*::*ADH1(t)*::*Kan^R^*	This study
pGF-IVL1169	pRS316; *prGAL*::*SpCas9*::*eGFP*::*NLS*::*NES*::*ADH1(t)*::*Kan^R^*	This study
pGF-IVL1170	pRS316; *prGAL*::*NLS*::*SpCas9*::*NLS*::*eGFP*::*ADH1(t)*::*Kan^R^*	This study
pGF-IVL1171	pRS316; *prGAL*::*NLS*::*SpCas9*::*NLS*::*eGFP*::*NLS*::*ADH1(t)*::*Kan^R^*	This study
pGF-IVL1172	pRS316; *prGAL*::*NLS*::*SpCas9*::*NLS*::*eGFP*::*NES*::*ADH1(t)*::*Kan^R^*	This study
pGF-IVL1173	pRS316; *prGAL*::*NLS*::*SpCas9*::*NLS*::*eGFP*::*NLS*::*NES*::*ADH1(t)*::*Kan^R^*	This study
pGF-IVL1174	pRS316; *prGAL*::*NLS*::*SpCas9*::*eGFP*::*ADH1(t)*::*Kan^R^*	This study
pGF-IVL1175	pRS316; *prGAL*::*NLS*::*SpCas9*::*eGFP*::*NLS*::*ADH1(t)*::*Kan^R^*	This study
pGF-IVL1176	pRS316; *prGAL*::*NLS*::*SpCas9*::*eGFP*::*NES*::*ADH1(t)*::*Kan^R^*	This study
pGF-IVL1177	pRS316; *prGAL*::*NLS*::*SpCas9*::*eGFP*::*NLS*::*NES*::*ADH1(t)*::*Kan^R^*	This study

a The *S. pyogenes* Cas9 gene was synthesized *de novo* (Genscript) with a yeast codon bias and assembled by *in vivo* ligation (Finnigan and Thorner 2015) under control of the *GAL1/10* promoter (814 bp 5′ UTR) and a C-terminal NLS (SRADPKKKRKV) signal sequence.

b The sgRNA cassette was synthesized *de novo* and modeled on previous work (DiCarlo *et al.* 2013; Finnigan and Thorner 2016). It contains 269 bp of the *SNR52* promoter and 20 bp of the 3′ UTR of *SUP4*. Various methodologies (*e.g.*, restriction digests and *in vitro* ligation) were used to subclone the sgRNA cassette from the original pUC57 vector to TOPO II (pCR-Blunt II-TOPO, Kan^R^-marked; Invitrogen) and to either pRS315 or pRS425 (or other pRS-family vectors). The (u2) guide sequence is 5′ GCTGTTCGTGTGCGCGTCCT 3′. For the sgRNA(u2) plasmid cloned into pRS423 (pGF-V798), the backbone sequence contains 317 bp of 5′ UTR and 201 bp of 3′ UTR flanking genomic sequence to the *HIS3* locus.

c The 20 bp (u1) guide sequence is 5′ CGGTGGACTTCGGCTACGTA 3′. For guide RNAs of 21 or 22 bp, the sequence included an additional GA inserted at the 5′ end.

d For sgRNAs of lengths <20 bp, the 3′ most segment of the target site was used.

e The mismatch(es) occur at the 5′ end of the sgRNA guide sequence. G/C was (randomly) changed to A/T and vice versa.

f The penultimate bp at the 5′ end of the sgRNA sequence was deleted.

g The NLS signal sequences used in pGF-IVL1162–pGF-IVL1177 are identical at the amino acid level, yet have codons altered at the DNA sequence level to aid in plasmid assembly. The central (between Cas9 and eGFP) NLS signal is immediately followed by a short flexible linker (SGSGS). The *S. pyogenes* Cas9 gene has a yeast codon bias.

h The NES signal (LAKILGALDIN) immediately follows the eGFP sequence. This sequence was modeled after the prototypical cyclic AMP-dependent protein kinase inhibitor NES (Wen *et al.* 1995; Kosugi *et al.* 2008).

i The C-terminal NES signal is separated from the penultimate NLS signal by two glycine residues.

### Culture conditions

Yeast were propagated in solid or liquid medium including YPD (2% peptone, 1% yeast extract, 2% dextrose) or synthetic media containing a nitrogen base, ammonium sulfate, and all necessary amino acid supplements. Preinduction medium included a 2% raffinose and 0.2% sucrose mixture. For experiments requiring induction of the *GAL1/10* promoter, liquid media containing 2% galactose was used. All sugars were filtered sterilized rather than autoclaved. The nomenclature for synthetic media is as follows: “S” refers to synthetic complete (*e.g.*, SD-URA, synthetic complete plus dextrose minus uracil).

### CRISPR-Cas9-based editing

The mCAL system (Finnigan and Thorner 2016) was used for all Cas9 editing in haploid yeast and in diploid gene drive–containing strains. Briefly, this system harnesses two artificial, programmed Cas9 target sequences (u1 and u2) that contain a maximum mismatch to the *S. cerevisiae* genome. Two identical (u1) sites flank the gene “target” locus whereas two identical (u2) sites flank the Cas9 gene cassette itself. Both the (u1) and (u2) sites within the genome also contain the 5′-NGG-3′ PAM required for *S. pyogenes* Cas9. All sgRNAs were designed to target either the (u1) or (u2) artificial sites. For plasmid-driven Cas9, the *URA3*-based vector (*e.g.*, pGF-IVL1116) was transformed into the appropriate yeast strain prior to editing for several reasons: (i) growth on dextrose repressed Cas9 expression, (ii) rapid counterselection of the vector could be achieved on media containing 5-FOA, and (iii) consistent propagation of the plasmid could be maintained prior to introduction of the sgRNA-expressing plasmid.

Activation of Cas9 and gene editing in haploid yeast was performed as previously described (Finnigan and Thorner 2016). Strains harboring the Cas9 vector were cultured overnight in a raffinose/sucrose mixture at 30° to saturation. Next, yeast were back-diluted into a YP + galactose mixture to an OD_600_ of ∼0.35 OD/ml and grown for an additional 4.5 hr. Cells were harvested and transformed with 1000 ng of sgRNA-containing plasmid, heat-shocked for 0.75 hr at 42°, incubated in YP + galactose overnight (∼16 hr) without shaking, spread onto SD-URA-LEU plates, and incubated for 3–4 d prior to imaging. The number of colonies was quantified using a single-blind protocol (researchers who were counting were unaware of the genotype of each plate) and a sectoring method (when appropriate). Several random fractions (one-quarter or one-eighth, *etc*.) were sampled and averaged to estimate the total number of colonies. For plates containing <500 colonies, the entire plate was quantified. To assess the drug resistance of individual colonies, 50–200 colonies were randomly selected and transferred to an identical plate type as a small patch (to increase the surface area) and incubated for 1–2 additional days. Next, plates (each containing between 50 and 100 colony patches) were replica-plated using sterile velvet cloths to rich medium containing dextrose and hygromycin (300 μg/ml) or G418 (200 μg/ml). Assessment of the *HIS3* locus was performed by reselecting clonal isolates, preparing purified chromosomal DNA, and PCR amplification and DNA sequencing (when appropriate).

### Gene drives and containment

Cas9 gene drives were constructed and manipulated using the following protocol. First, the Cas9-expression cassette was integrated at the *HIS3* locus and maintained on dextrose (to repress the *GAL1/10* promoter). Second, yeast were transformed with the sgRNA(u1) plasmid; since the target (u1) sequence does not exist within *S. cerevisiae*, editing is halted even if Cas9 was present and primed with the guide RNA. Third, haploid yeast expressing the sgRNA were mated to the gene “target” containing strain (harboring (u1) sites) of the opposite mating type on rich media containing dextrose for 24 hr at 30°. Fourth, yeast were transferred to SD-LEU-HIS plates using sterile velvet cloths to select for diploid yeast and incubated for 24 hr at 30°. The diploid selection step was repeated a second (or sometimes third) time on the same media type. The choice of the *Schizosaccharomyces pombe HIS5* gene within the target genome to select for diploids ensures any (rare) promiscuous Cas9 that may have prematurely been activated will be destroyed on SD-LEU-HIS medium. Fifth, diploid yeast were cultured overnight in synthetic medium containing raffinose, sucrose, and lacking leucine to saturation. Sixth, cells were back-diluted to an OD_600_ of ∼0.35 OD/ml in YP medium containing galactose for 0–24 hr at 30°, depending on the time course. Following induction of Cas9, yeast were harvested, washed with water once, and diluted to a density of ∼1000 cells/ml. Between 250 and 1000 cells were spread onto SD-LEU plates and incubated for 2–3 d. Yeast were transferred to a fresh SD-LEU plate and SD-HIS plate using sterile velvet cloths and incubated for 24 hr prior to imaging. The number of colonies sensitive on SD-HIS medium was quantified (over total number of colonies on SD-LEU) to obtain the percentage of active drives within a given genotype. Analysis of individual colonies was done by reselecting yeast on SD-LEU medium and testing individual clonal isolates for growth on various media types, ploidy status (by mating to *MAT***a** and *MAT***⍺** control strains), and chromosomal DNA isolation for diagnostic PCRs and DNA sequencing. For gene drives harboring two plasmids (*URA3*/*LEU2*-marked), the procedure included diploid selection on SD-URA-LEU-HIS plates, and final testing on SD-URA-LEU to maintain the presence of both plasmids.

A number of safeguards were included to ensure the safe, ethical, and contained use of all yeast strains harboring the (potentially) active CRISPR gene drive arrangement. First, the most powerful safeguard includes the use of programmed target DNA site (u1) at the *HIS3* locus that does not exist within the native budding yeast genome (Finnigan and Thorner 2016). This sequence has a maximum mismatch to any other sequence within *S. cerevisiae* reducing the possibility of inappropriate editing (off-target effects) and virtually eliminating the possibility of the drive to propagate within a wild yeast population of any strain type or related species. Second, all haploid gene drive strains [containing Cas9 and the (u1)-targeting sgRNA plasmid] were grown on dextrose to repress transcription of Cas9. Within the diploid strain, Cas9 was only activated for a limited amount of time (0–24 hr). Third, the sgRNA to target the (u1) sequence was exclusively maintained on a high-copy yeast plasmid (pRS425). Previous work (DiCarlo *et al.* 2015) has suggested that separation of the guide sequence from the Cas9 gene can provide an additional safeguard. Here, we have also documented the rapid loss of sgRNA(u1) plasmid from diploid yeast in the absence of any selective pressure (Supplemental Material, Figure S5 in File S1). Fourth, all the Cas9-expression cassettes are also flanked by the artificial (u2) site. This serves as a specific safeguard to exactly excise the entire Cas9 gene and associated sequence from any haploid or diploid genome in a single step. We demonstrate that introduction (by direct transformation or mating via a strain of the opposite mating type) of the (u2) sgRNA plasmid causes removal and destruction of the existing drive system (Figure S6 in File S1). All gene drive strains were constructed with this (u2) system in place. Fifth, the laboratory diploid strain (BY4741/BY4742) has been previously documented to be very inefficient at sporulation, even under optimal conditions that induce meiosis and spore formation (Heasley and McMurray 2016). Sixth, careful destruction of all haploid and diploid yeast immediately following experimentation was performed (including capture of all washes of glassware for autoclaving). All materials used (tubes, velvet cloths, pipette tips, plates, wooden sticks, liquid cultures, *etc*.) were autoclaved at >121° for at least 0.75 hr before disposal or reuse. Diploid yeast strains containing gene drive systems were all destroyed (not frozen) after experimentation.

### Fluorescence microscopy

Yeast were grown overnight in a preinduction culture containing raffinose and sucrose to saturation and back-diluted into YP + galactose for 4.5 hr. Cells were harvested, washed with water, prepared on a microscope slide with a coverslip, and imaged within 15 min. Yeast were imaged on an inverted Leica DMI6500 fluorescence microscope (Leica Microsystems Inc., Buffalo Grove, IL) with a 100× objective lens, and fluorescence filters (Semrock, GFP-4050B-LDKM-ZERO and mCherry-C-LDMK-ZERO). A Leica DFC340 FX camera, Leica Microsystems Application Suite AF software, and ImageJ (National Institutes of Health) were used to process all images. All images were obtained using identical exposure times and were processed and rescaled together. The “merged” images were generated in ImageJ and do not contain any additional processing from the GFP/mCherry images. The yeast cell periphery (and the yeast vacuole) was determined using the DIC image. Representative cells were chosen for each image.

### Data and reagent availability

Upon request, we will freely send renewable research materials (yeast strains and plasmids) and reagents that were created during the course of this study for research purposes (not-for-profit institutions). The exception includes diploid yeast strains harboring active (guide RNA plasmid) gene drive configurations (all were destroyed)—haploid parental strains will be provided instead.

## Results

### An artificial system for Cas9-based gene editing in budding yeast

**Figure 1:**
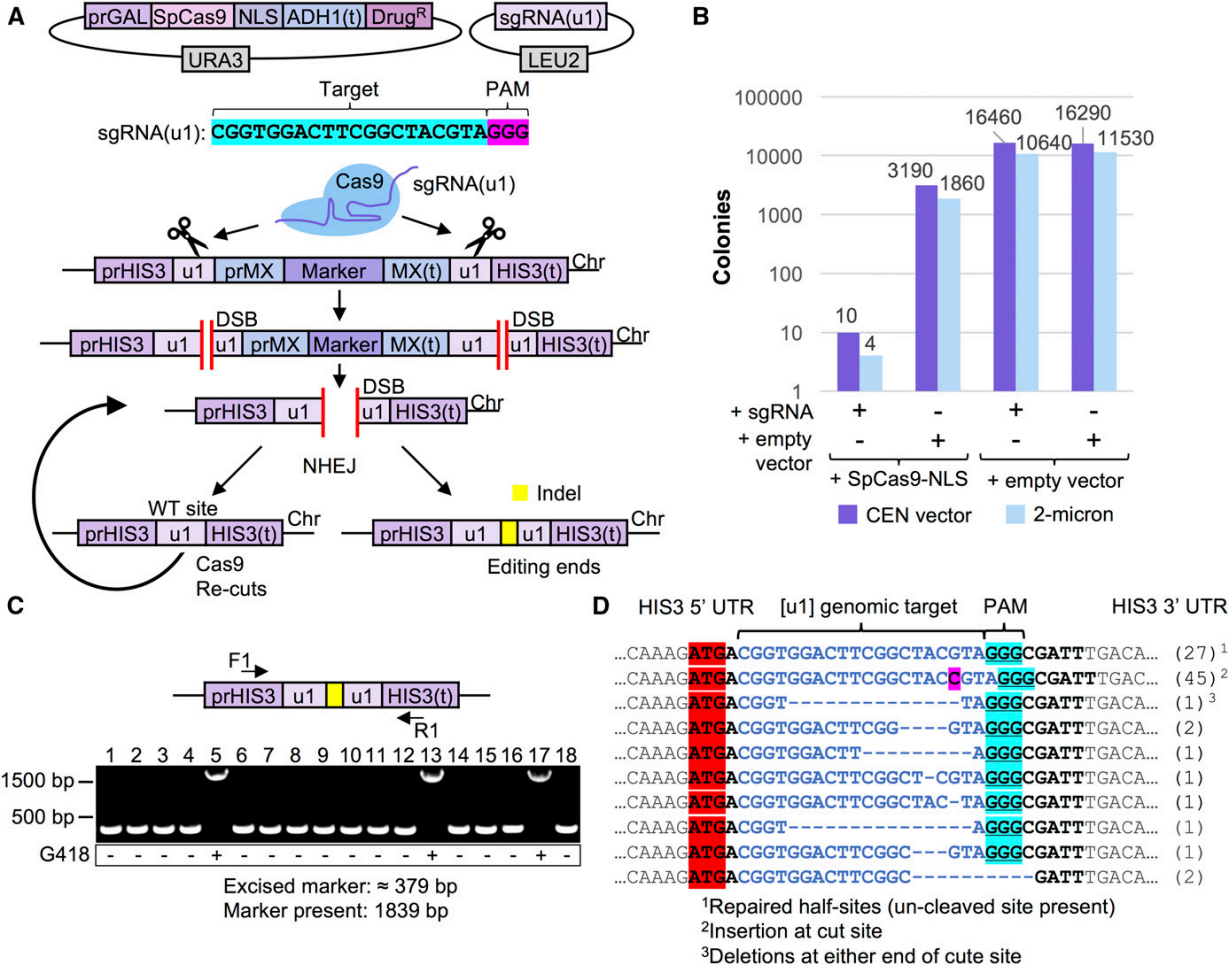
A safe, programmable system to test CRISPR-based gene editing in haploid yeast. (A) Our design for a yeast system for analysis of CRISPR editing includes (i) an inducible *S. pyogenes* Cas9 expressed from a *URA3*-based plasmid, (ii) a sgRNA expression cassette on a high-copy *LEU2*-based plasmid, and (iii) a programmable gene “target” (consisting of a drug resistance marker cassette) at a safe-harbor locus (*HIS3*) flanked by two “unique” DNA sequences (u1) that do not exist within the *S. cerevisiae* genome (Finnigan and Thorner 2016). Induction of Cas9 allows targeting and double-stranded break formation at the identical (u1) sequences. In the absence of exogenous DNA (*e.g.*, amplified PCR product) to be used for HDR, NHEJ of the exposed chromosomal ends causes full excision of the selectable marker. However, given the unique arrangement of the identical (u1) sites, NHEJ in the absence of any insertion/deletion mutation at the Cas9 cut site (left) recreates another WT (u1) site and subsequent re-editing of the same target sequence until Cas9 expression is shutoff or a mutation is positioned within the (u1) site (right). (B) Cas9-dependent editing results in cell inviability. GFY-2353 yeast already harboring Cas9-NLS on a vector (pGF-IVL1116) or an empty vector control (pRS316) were induced in medium containing galactose, transformed with the sgRNA(u1)-expression cassette on either a CEN-based (pGF-V1215) or 2μ-based (pGF-V1220) plasmid, and plated onto SD-URA-LEU media. (C) GFY-2588 yeast containing pGF-IVL1342 were transformed with sgRNA(u1) plasmid (pGF-V1216) and selected on SD-URA-LEU medium. The isolated chromosomal DNA of individual clonal (surviving) isolates was assayed by PCR using DNA oligonucleotides (F1/R1, Table S1 in File S1) to the flanking *HIS3* UTR. The expected product sizes of the amplified PCR fragments are ∼379 bp (depending on the type of insertion/deletion(s) at the cut site, if any), or 1839 bp in the absence of any editing. Colonies were tested for resistance on medium containing G418 (below). (D) Clonal isolates from Cas9 editing (a dozen independent experiments) using the high copy sgRNA(u1) plasmid from (B) and that had also excised the selection cassette were analyzed by DNA sequencing at the *HIS3* locus. The number of each genotype obtained is listed (right).

The goal of this study was to identify aspects of CRISPR gene editing that have the potential to modify, in a predictable manner, the activity and effectiveness of gene drives. We envision that the molecular mechanisms we identify and study would have direct relevance and application to CRISPR drive systems in other organisms for various reasons. Given the recent interest and potential applications of nuclease-based gene drives across numerous industries, we sought to develop a safe, programmable system in *S. cerevisiae* to investigate control of Cas9 editing *in vivo*. Our methodology includes several unique aspects that (i) remove concerns of off-target effects, (ii) minimize the number of sgRNA constructs required, (iii) allow for an unbiased assessment of editing (no selective pressure for or against an editing event), (iv) allow for full excision of a target marker (rather than rely on disruption of a coding sequence by a single targeted event), and (v) provide a potent genetic safeguard for use within a gene drive system (Figure 1A). We developed a “target” strain of yeast flanked by two unique artificial sequences, termed (u1). We have previously described use and application of placement of these unique sites throughout the genome to allow for multiplexing with only one sgRNA construct and Cas9 (Finnigan and Thorner 2016). Since these programmed (u1) sequences do not exist within the yeast genome (and provide a maximum mismatch to the closest native sequences), this reduces any possibility of off-target effects, or biased results based on similar gene target(s) or repetitive sequences. Moreover, our design includes two (u1) sites flanking a selectable marker (conferring hygromycin or G418 resistance). This is in stark contrast to numerous other Cas9 editing assays (DiCarlo *et al.* 2013, 2015), which target the coding sequence of a given gene (or marker) and depend upon disruption of the final protein product to provide a detectible growth phenotype. This approach includes several issues that might arise from “traditional” targeting with a single sgRNA construct, opposed than our system of a “clean” excision of the entire physical gene. First, since disruption of the gene (*e.g.*, *CAN1*) product allows for growth (on medium containing canavanine), there would be selective pressure to perform either NHEJ or HDR and allow for cell survival. Second, while traditional red/white colony color screening has been a very useful genetic tool for screening (adenine biosynthesis), the presence of the red pigment is slightly toxic to cells (Ugolini and Bruschi 1996). Third, editing events that faithfully repair the DNA break without including an insertion or deletion would be virtually undetectable as there would be no change in sequence following repair via NHEJ. Fourth, use of two distinct sgRNAs to target two positions at a genomic locus of interest would require different target sequences, and this may allow for preference or bias to one cut site. Our novel system controls for any difference in target sequence since all targets are identical (and allow for up to 22 bp targeting with a 3 bp PAM); this is the first setup to provide two identical Cas9 cut sites flanking a selectable marker that imposes no native selective pressure for or against said marker, since selection for the target is not assayed until after the editing event. This is an important technical difference between our system and traditional Cas9 editing approaches. Successful targeting of Cas9 to both sites, introduction of two DSBs, and subsequent repair of the broken DNA by NHEJ would recreate a single (u1) site from fusion of the two flanking “half sites,” along with full excision of the marker gene (Figure 1A). In this way, we would be able to detect Cas9 editing of a single sequence in the absence of any generated indel by NHEJ.

Yeast containing our (u1) flanked target gene and an inducible *S. pyogenes* Cas9 (*GAL1/10* promoter) were transformed with either control (empty) plasmids or sgRNA(u1)-expressing plasmids (low or high copy) in the presence of galactose (Figure 1B). Importantly, the subsequent selection step following introduction of the guide RNA plasmid and activation of Cas9 (located on a separate plasmid) was for the presence of both vectors. In the absence of any donor DNA fragment to repair the edited *HIS3* locus, yeast activate the NHEJ pathway and repair the cleaved DNA together (without our selectable marker). In the presence of both the sgRNA(u1) guide and active Cas9, only a small number of surviving colonies were obtained; this is consistent with previous studies in budding yeast using *S. pyogenes* Cas9 (DiCarlo *et al.* 2013; Finnigan and Thorner 2016). Following introduction of a DSB, the NHEJ system allows for precise repair of the severed (u1) sites. However, NHEJ with no alteration of sequence would result in a functional target (u1) DNA site and a second round of Cas9-dependent cleavage. Consecutive rounds of editing followed by repair occur during treatment with galactose and, given the excess of Cas9 available per cell, results in few cells that are viable once plated onto selective media. (Figure 1B). In this way, general cell viability serves as a potent selection mechanism for assaying Cas9 editing in yeast. When individual surviving clones were tested for drug resistance following editing, the majority had fully excised the gene cassette as assayed by diagnostic PCRs of the *HIS3* locus (Figure 1C). Finally, DNA sequencing of a large pool of isolated clones yielded a diverse assortment of insertions and deletions at the “repaired” (u1) site following editing by Cas9 (Figure 1D). Many clones (27 separate isolates) did not contain any alteration at the expected Cas9 cut site (+3 position upstream of the 5′ end of the PAM). However, this was not due to a lack of editing because the entire drug selection marker was not present and had been fully excised. Interestingly, plating of yeast onto galactose containing medium (rather than dextrose) following transformation of the sgRNA plasmid resulted in a 10-fold decrease in the number of surviving colonies (Figure S1 in File S1). Moreover, none of the surviving clones had excised the selectable marker or included any indel at the (u1) site(s), suggesting that prolonged Cas9 activation increases the stringency of selection. Thus, we have provided the first evidence to suggest that, in fact, estimates of editing are limited to detection of a generated indel at the proposed editing site. Our arrangement clearly demonstrates the ability of NHEJ to repair cleaved DNA with no alteration of sequence, at least in budding yeast. Additionally, our assay for Cas9 editing does not impose selection for any altered genetic marker until after the editing event, prevents off-target effects, and can also achieve multiplexing using identical sequences preprogrammed within a genome and an sgRNA fragment.

### Exploring control of CRISPR editing in haploid cells

**Figure 2:**
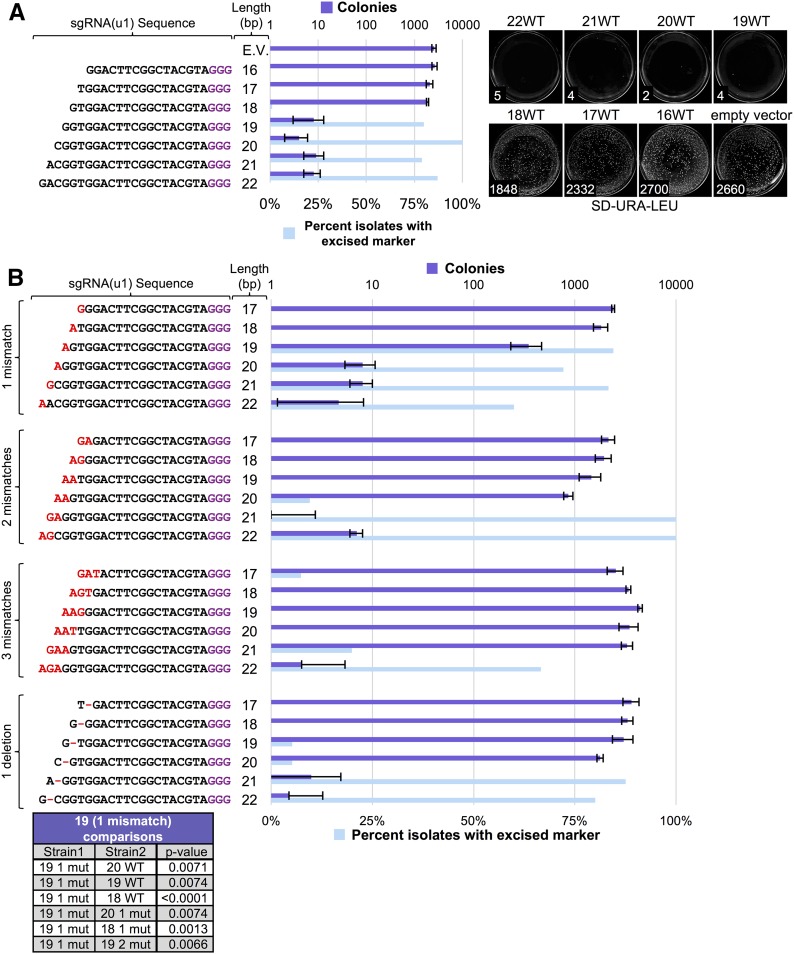
Effect of sgRNA length and 5′ target mismatch on Cas9 editing efficiency. (A) GFY-2353 yeast containing the Cas9-NLS vector (pGF-IVL1116) were transformed with sgRNA(u1) cassettes (plasmids pGF-V1216–pGF-V1222) with guide sequences of varying length along with an empty pRS425 vector control. The number of colonies was quantified (left) for three independent trials. Error, SD. Representative plates are shown (right). A random sampling of colonies was chosen across all three trials following editing on SD-URA-LEU plates and tested for growth on rich medium containing hygromycin. The percentage of isolates displaying sensitivity to the drug were quantified. For conditions (*e.g.*, sgRNA(u1) 20 bp length) where a small number of colonies were viable, all surviving isolates (typically 5–20 total) were tested on hygromycin; for other combinations, between 150 and 200 colonies were sampled. (B) Cas9 editing was repeated as in (A) using sgRNA(u1) cassettes containing varying mismatches at the 5′ end of the guide sequence. A single mismatch at the 5′ end (pGF-V1223 – pGF-V1228), two mismatches (pGF-V1229 – pGF-V1234), three mismatches (pGF-V1235 – pGF-V1240), or a deletion of one base at the penultimate −2 position from the 5′ end (pGF-V1241–pGF-V1246) were assayed for both total surviving colonies and the percentage of isolates with an excised marker cassette at the target locus (top). Select comparisons with the sgRNA(u1) 19 bp guide with one mismatch data were performed using an unpaired *t*-test (bottom).

Since our long-term goal is the exploration of gene drive control and its possible application in plants, fungi, microbes, and metazoans, we limited our focus to the conserved components of the CRISPR system, namely, the Cas9 nuclease and the sgRNA. While additional yeast-based (or fungal-based) assays might be explored in the future, our objective was to investigate common components of all gene drive systems. Given that previous studies have analyzed the contribution of sgRNA identity, GC content, length, mismatch, stem-loop structure, and other modifications (Dang *et al.* 2015; Xu *et al.* 2015; Doench *et al.* 2016; Nowak *et al.* 2016; Zhang *et al.* 2016; Numamoto *et al.* 2017), we tested a set of sgRNAs variants within our yeast editing system (Figure 2). Of note, we have maintained an identical target DNA sequence [namely, the (u1) artificial target sites] in order to directly compare modifications to each guide sequence. However, we recognize that others (Hsu *et al.* 2013; Zheng *et al.* 2017) have demonstrated that the effect of sgRNA mismatches on targeting varies between sgRNAs. We first altered the guide length from 16 to 22 bp and found that editing in our system required a crRNA length between 19 and 22 bp (Figure 2A). Next, for guide lengths of 17–22 bp, we varied the number of 5′ mismatches (one, two, or three consecutive mutations: A/T to G/C and vice versa, chosen at random) as well as deletion of the penultimate base at the 5′ end (Figure 2B). Testing of isolated clones for excision of the selectable marker largely mirrored the cell viability results—guide RNAs allowing efficient Cas9 editing resulted in loss of the marker whereas a lack of or reduction in editing was paired with drug resistance. Our results indicate that sgRNA length and identity had either no effect on editing or resulted in a complete loss of editing with only a single exception, a guide length of 19 bp with a single mismatch at the 5′ end. Numerous independent trials found an intermediate level of editing for this sgRNA to the dual (u1) sites. A total of 85% of yeast clones had excised the drug resistance cassette, yet editing with this sgRNA always resulted in more surviving colonies. We suspect that, given the unique arrangement of our (u1) flanked system, repair of the broken chromosome ends results in presentation of a (third) wild-type (WT) (u1) target site that can also be edited by Cas9 (Figure 1A). A slight reduction in Cas9 targeting may impede this editing and “re-editing” cycle to allow an increase in surviving yeast clones. However, altering the 5′ mismatch from G→A to either G→C or G→T resulted in a near loss of editing and a phenocopy of the 18 bp guide length sgRNA (Figure S2 in File S1). These results suggest editing using the 19 bp guide length with a 5′ mismatch is likely sequence and/or context dependent. We also observed a similar trend for other sgRNA variants that included mismatches. For instance, a 20 bp guide with two mismatches at the 5′ end provided a slight reduction in the total number of colonies (compared with the 18 bp guide length with no mismatch) and a small fraction of colonies that had properly excised the drug marker. While our analyses of sgRNAs is by no means comprehensive, our results demonstrate that all but one guide sequence (32/33 tested) either allow for optimal editing, or do not allow for editing at all, with potentially only a few rare exceptions (such as the 19 bp guide with one 5′ mismatch) that may result in an intermediate level of editing.

**Figure 3:**
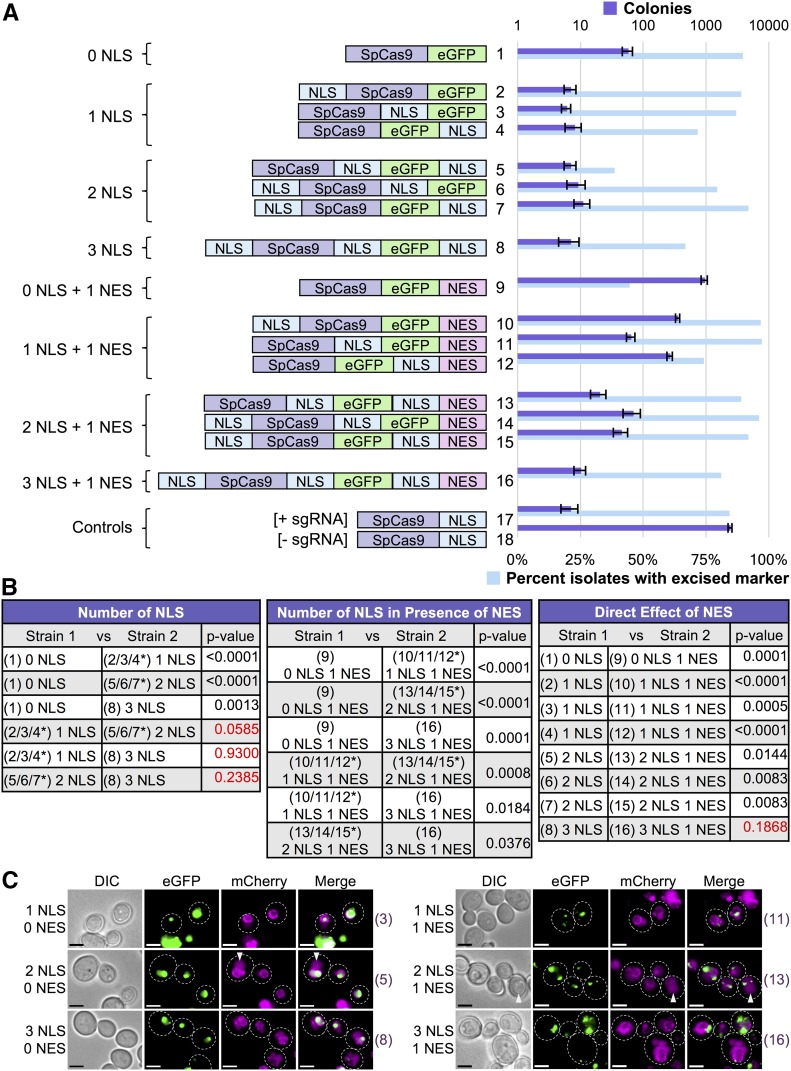
Nucleocytoplasmic shuttling of Cas9 to control gene editing. (A) 16 variations of Cas9-eGFP were constructed that included combinations of NLS and/or NES signals at various protein positions. Either 0, 1, 2, or 3 (identical) NLS signals were included along with either 0 or 1 NES signals; the positions chosen included the N-terminus, between Cas9 and eGFP, or at the C-terminus (left). GFY-2353 yeast were transformed with each Cas9 fusion (pGF-IVL1162 – pGFIVL1177) along with Cas9-NLS (pGF-IVL1116) as a positive control. Editing was performed by induction of Cas9 expression followed by transformation of equimolar amounts of sgRNA(u1) (20 bp WT guide) plasmid in triplicate. The strain expressing Cas9-NLS served as a control (transformed with either sgRNA(u1) or an empty pRS425 vector). The total number of surviving colonies (SD-URA-LEU medium) was quantified. Error, SD. Following editing, randomly selected isolates from all trials (*n* = 100–200) were tested for growth on rich media containing hygromycin. For combinations where only a few surviving colonies existed, all possible isolates were tested for hygromycin sensitivity. (B) Comparisons of the colony counts between two strains from (A) were analyzed using an unpaired *t*-test. Red text indicates p-values > 0.05. (C) Six Cas9-eGFP fusions were integrated into the yeast genome at the *HIS3* locus in a strain expressing an endogenously tagged Nup188-mCherry to mark the nuclear periphery (strains GFY-3264–GFY-3267, GFY-3273, and GFY-3275). Cultures were induced in galactose for 4.5 hr prior to imaging by fluorescence microscopy. Scale bar, 3 μm. White dotted lines, cell periphery. White triangles, yeast vacuole. Strain numbers (right) refer to the Cas9 fusions in (A) for clarity.

Next, we investigated whether nucleocytoplasmic shuffling might be used to control Cas9 editing. Recently, a Cas9 variant (iCas) used occlusion of an NLS signal to prevent entry into the nucleus until treatment with an added external chemical (Liu *et al.* 2016). We hypothesized that nuclear localization and residence time might provide a conserved means to control and titrate editing when all other factors (Cas9 protein, sgRNA, target DNA) are held constant. We constructed a C-terminal Cas9-eGFP fusion and confirmed that it was fully functional and competent for editing compared with Cas9 alone (Figure S3 in File S1). This provided three locations (N-terminus, C-terminus, and between the Cas9 and eGFP fusion) onto which to include an NLS sequence. We tested eight variants of Cas9 containing zero, one, two, or three NLS signals in all possible combinations and their ability to edit *in vivo* (Figure 3, A and B). A Cas9-eGFP (lacking any added NLS) variant was still able to edit, albeit at a lower level compared with all other Cas9-eGFP variants harboring at least one NLS sequence. We suspect that Cas9 itself may (i) harbor a cryptic NLS signal(s) consisting of a cluster of positively charged residues and/or (ii) achieve a low level of diffusion into the nucleus, despite its large molecular weight (Wang and Brattain 2007). There is precedence for unintended (artificial) nuclear localization of exposed peptides or nonnative protein sequences (Guo *et al.* 2011; Finnigan *et al.* 2016). We did not observe any significant differences in this assay for all Cas9 fusions containing only one to three NLS signals (one NLS was sufficient to promote maximum editing), although others (Ménoret *et al.* 2015; Torres-Ruiz *et al.* 2017) have reported increases in editing following the addition of numerous NLS sequences in other cell systems.

Additionally, we directly fused a C-terminal NES signal sequence to each of the eight Cas9-NLS variants to determine if the interplay between import and export shuttling would affect editing (Figure 3, A and B). Our results demonstrate that the direct competition between nuclear export and import can titrate the level of gene editing in otherwise isogenic strains. Direct fusion of an NES signal to the Cas9-eGFP variant greatly reduced editing, yet did not fully eliminate nuclear access (compare fusions 1 with 9) because nearly 50% of surviving clones had properly excised the target drug resistance marker (Figure 3A). Furthermore, this supported the observation that our Cas9-eGFP could access the nucleus in the absence of any added NLS signal. Direct competition of one NLS *vs.* one NES signal had the strongest reduction in gene editing (compare fusions 2/3/4 with 10/11/12). Altering the competition to two NLSs *vs.* one NES shifted the level of editing (compare fusions 5/6/7 with 13/14/15). Finally, a Cas9 variant harboring three NLS signals and one C-terminal NES displayed WT levels of editing (compare fusions 2/3/4 with 16). Live cell imaging of strains harboring one, two, or three NLS were compared with the same constructs also containing an NES (Figure 3C). The nuclear periphery was marked with Nup188-mCherry; steady-state levels of Cas9-eGFP were found within the nucleus for fusions containing only NLS signals (Figure 3C, left) whereas the presence of the NES signal caused spatial exclusion from the nucleus (Figure 3C, right).

These data demonstrate that, given sub-optimal nuclear localization (via the presence of an appended NES signal) there is a definite contribution of having more than one NLS sequence present. In our yeast system, given (i) sufficient *GAL1/10*-driven expression of Cas9 and (ii) an extended recovery phase overnight in medium containing galactose, the SV40 NLS sequence was sufficient to promote maximal editing (Figure 3A). Therefore, in other cell systems where the SV40 may represent a nonoptimal signal sequence, or a lower level/amount of Cas9 protein present, it appears the addition of extra nuclear localization signals can improve import and subsequent editing.

**Figure 4:**
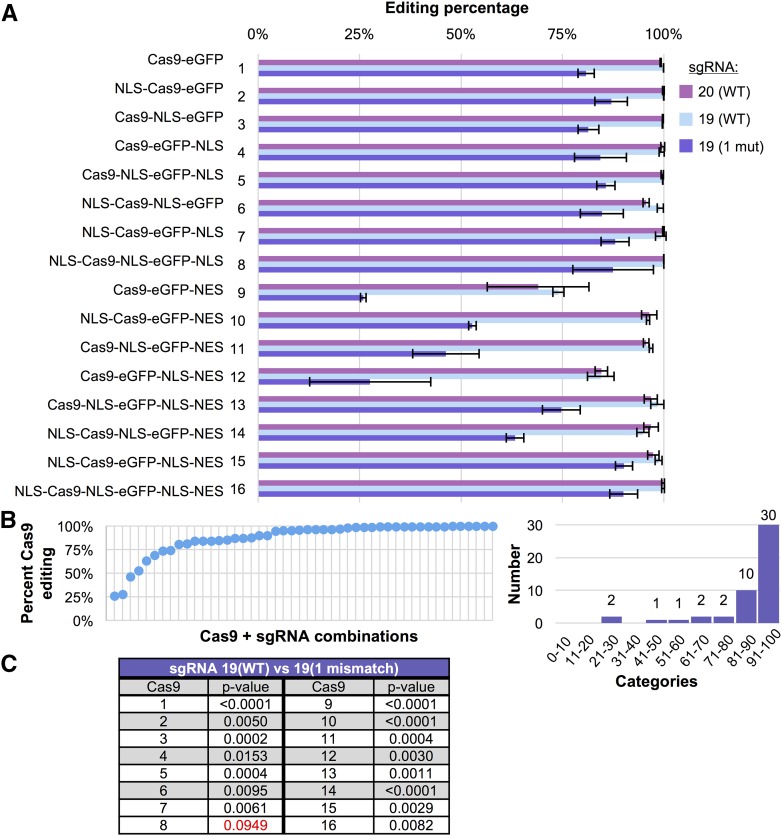
Editing of haploid yeast using a combination of sgRNA mismatch and Cas9 nuclear localization. (A) GFY-2353 yeast containing 16 Cas9-eGFP fusions with NLS/NES combinations (pGF-IVL1162–pGF-IVL1177) from Figure 3 were transformed with sgRNA(u1)-expressing plasmids (pGF-V1219, pGF-V1220, and pGF-V1225) or empty pRS425 and the total number of colonies quantified. For each Cas9-eGFP fusion, all four plasmids were transformed in triplicate. The editing efficiency is displayed as a percentage by the following calculation: (i) the total number of colonies (per sample) was first divided by the total number of colonies obtained for the empty vector control followed by (ii) 100% minus the calculated percentage from (i). Error, SD from (i). (B) The data from (A) is displayed from lowest to highest editing percentage (left) or as a histogram with 10% binning categories (right). (C) Select comparisons (sgRNA(u1) 19 WT *vs.* 19 with one mismatch) between editing percentages from (A) were analyzed using an unpaired *t*-test. Red text, p-values > 0.05.

Finally, we examined whether there would be an additive effect between sgRNA identity (Figure 2) and nuclear shuttling (Figure 3) by testing each of the Cas9-eGFP fusions with three different sgRNAs (20 bp WT, 19 bp WT, and 19 with one 5′ G→A mismatch) (Figure 4). By comparing the number of yeast colonies for each of the guide RNAs (editing) to the number of colonies for an empty sgRNA vector (control) transformation, we determined a standardized “editing percentage.” For combinations of Cas9 and sgRNA that resulted in zero colonies, the editing percentage would be 100%. Conversely, a combination with 1000 colonies where the empty vector control also yielded 1000 colonies would calculate as 0% editing. As expected, the eight Cas9 fusions lacking any NES signal provided nearly 100% editing. Use of the sgRNA with a 19 bp guide and single 5′ mismatch reduced the editing by ∼15–25% (Figure 4A). Testing of the Cas9 fusions in the presence of a C-terminal NES signal displayed the same pattern (Figure 3) for both 20 bp (WT) and 19 bp (WT) sgRNAs—a reduction in editing based on the number of competing NLS signals present. Use of the 19 bp guide RNA with a single mismatch caused a significant reduction (15–75%) in editing that was also correlated to the number of NLS signals, as more NLS sequences buffered against the effects of the less-effective guide RNA. Additionally, we observed the Cas9-eGFP-NLS-NES construct (fusion 12) deviated from the other two single NLS *vs.* single NES variants (fusions 10 and 11). In our experiments, the NLS-NES tag (fusion 12) nearly phenocopied the NES tag alone (fusion 9). Given the immediate proximity of the two signals (separated by only two residues), we suspect that physical access to the penultimate NLS might be restricted by binding and competition by nuclear export machinery (and possibly vice versa as well). We demonstrate using these 48 combinations of Cas9 fusions and sgRNAs a wide range of editing efficiencies can be achieved with the majority between 75 and 100% effectiveness (Figure 4B). Moreover, our data illustrate there is a statistically significant added alteration in editing when using the 19 bp guide RNA with a single 5′ mismatch across 15 out of 16 of our Cas9 fusions with the only exception being Cas9-eGFP with a triple NLS signal (Figure 4C).

### Four independent mechanisms to titrate gene drive activity

**Figure 5:**
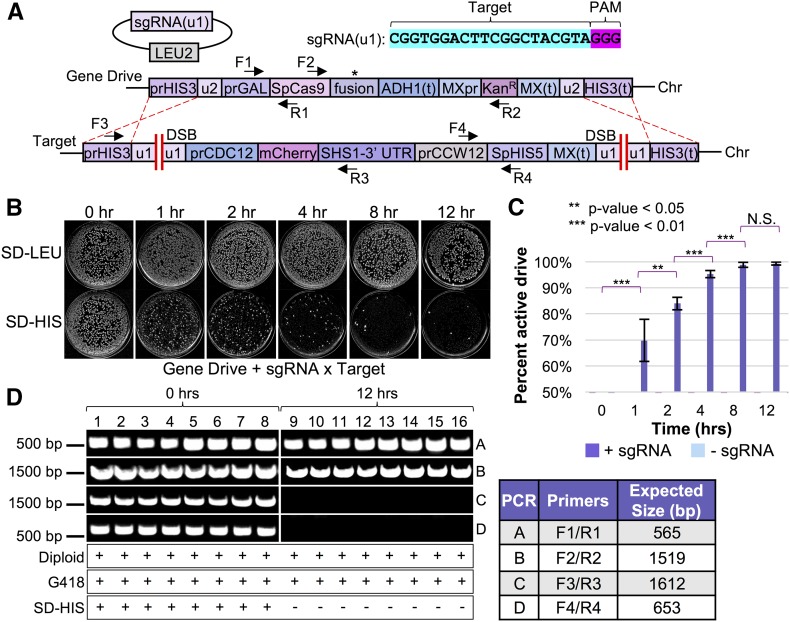
Altering levels of Cas9 to activate an artificial gene drive in diploid yeast cells. (A) Our design of a programmable gene drive included (i) an integrated copy of *S. pyogenes* Cas9 (asterisk denotes use of various Cas9 fusions in an otherwise identical construct) under the inducible *GAL1/10* promoter at the *HIS3* locus in *MAT*a cells, (ii) a Kanamycin-resistance gene cassette, (iii) flanking unique sites (u2) (Finnigan and Thorner 2016) surrounding the entire gene drive system to be used as a genetic failsafe (see Figure S6 in File S1), and (iv) an artificial gene “target” containing a different selectable marker (*S. pombe HIS5*) and flanked by (u1) artificial Cas9 sites at the *HIS3* locus in a strain of the opposite mating type (*MAT*α). (B) Activation and testing of all gene drives was performed as follows. First, the Cas9-containing strain (shown, GFY-2383) was transformed with the sgRNA(u1) plasmid (pGF-IVL1220) or an empty vector (pRS425) control and maintained on dextrose. Second, the gene drive strain (*MAT*a) harboring the sgRNA(u1) plasmid was mated to the target strain (*MAT*α; GFY-3206 or GFY-3207) on rich medium for 24 hr at 30°. Third, diploid yeast were selected twice on SD-LEU-HIS medium (24 hr incubation at 30°). Fourth, diploids were cultured overnight in S-LEU + Raffinose/Sucrose liquid medium. Fifth, strains were back-diluted to an OD_600_ of ∼0.35 OD/ml in YP + Galactose and grown at 30° for various amounts of time. Sixth, yeast were harvested by a brief centrifugation, washed with water, diluted to ∼1000 cells/ml, and 0.5 ml was plated onto SD-LEU medium and incubated at 30° for 2 d. Finally, yeast were transferred by replica-plating to SD-LEU and SD-HIS plates and incubated for 24 additional hours before imaging. Representative plates are shown for the GFY-3206 cross. (C) Quantification of the percentage of colonies displaying an active gene drive (assayed by sensitivity on SD-HIS medium). Error, SD. Statistically significant comparisons are denoted using an unpaired *t*-test. N.S., not significant. The value for 0 hr is 0% drive activity, not 50%. Experimental runs with an empty plasmid (pRS425) were also performed and displayed a value of zero drive activity for all time points. (D) Clonal isolates were randomly selected from SD-LEU plates from (B) and retested on G418 and SD-HIS media. Multiple crosses were used to determine ploidy status. Diagnostic PCRs (A–D) were performed on isolated diploid chromosomal DNA to assess the *HIS3* locus at 0 and 12 hr post galactose shift (also see Figure S4 in File S1). Oligonucleotides used can be found in Table S1 in File S1.

Based upon our design for haploid gene editing, we construed a safe and programmable gene drive system in budding yeast (Figure 5A). Our drive strain included Cas9 under the inducible *GAL1/10* promoter, a drug resistance cassette (Kan^R^), and flanking artificial (u2) sites (as a genetic safeguard, see *Materials and Methods* and Figure S6 in File S1). For biosecurity reasons, we have kept the sgRNA-expression cassette separate from the physical chromosomal gene drive and, instead, maintained it on a high-copy plasmid (see *Materials and Methods*, Figure S5 in File S1). We generated an artificial gene “target” strain in yeast of the opposite mating type containing flanking (u1) sites at the *HIS3* locus including (ii) a target gene to be excised and (ii) a distinct selection cassette (*S. pombe HIS5*) under control of the constitutive *CCW12* promoter. Importantly, we included unique terminator sequences (*ADH1* and *SHS1* 3′ UTR) for both the drive and target systems; the presence of any identical sequences between the two homologous chromosomes can allow for inappropriate crossover within the drive itself (E. Roggenkamp, R. M. Giersch, M. N. Schrock, E. Turnquist, M. Halloran, and G. C. Finnigan, unpublished results).

The gene drive strain was transformed with the sgRNA(u1) plasmid (20 bp guide WT), mated to the target strain, and diploids were selected while maintaining growth on dextrose to repress Cas9 expression. Cultures were shifted to galactose for a time course between 0 and 12 hr and plated onto SD-LEU medium. Additionally, activity (or lack thereof) of the drive was not necessary for cell survival. In this way, we provided an unbiased assessment of the proportion of cells within a given sampling that were able to (i) express Cas9, (ii) bind sgRNA, (iii) target the dual (u1) artificial sequence(s), and (iv) undergo homologous recombination to repair the DSB and copying the entirety of the drive to the opposite chromosome to replace the target gene. Like our experimental design for assaying Cas9 activity in haploids (Figure 5A), there is no selective pressure of any kind during the editing and repair events. Once single colonies were sufficiently grown, the entire sampling was transferred to both SD-LEU and SD-HIS plates where we assessed the status of the *HIS3* locus gene drive and/or target. 100% of colonies maintained G418 resistance regardless of whether an active drive was induced (E. Roggenkamp, R. M. Giersch, M. N. Schrock, E. Turnquist, M. Halloran, and G. C. Finnigan, unpublished results). However, as cells were grown in galactose for increasing amounts of time, causing higher expression of Cas9, a larger proportion of the population was sensitive on the SD-HIS condition (Figure 5, B and C). After 4 hr postgalactose shift, 99% of colonies had lost the *S. pombe HIS5* marker within the target locus. A random sampling of clonal isolates (all tested and confirmed as diploids) from each time point were chosen from SD-LEU plates and the *HIS3* locus was interrogated by multiple diagnostic PCRs (Figure 5D and Figure S4 in File S1). There was a 100% correlation between colonies sensitive on SD-HIS medium and lack of the entire target locus as assayed by PCR. For those (few) surviving colonies on SD-HIS (even after the 12 hr galactose shift), the diploid genome still contained both the Cas9 gene drive and the target cassette (E. Roggenkamp, R. M. Giersch, M. N. Schrock, E. Turnquist, M. Halloran, and G. C. Finnigan, unpublished results). These data suggest that titration of the Cas9 nuclease itself correlates strongly with gene drive activity within a population.

**Figure 6:**
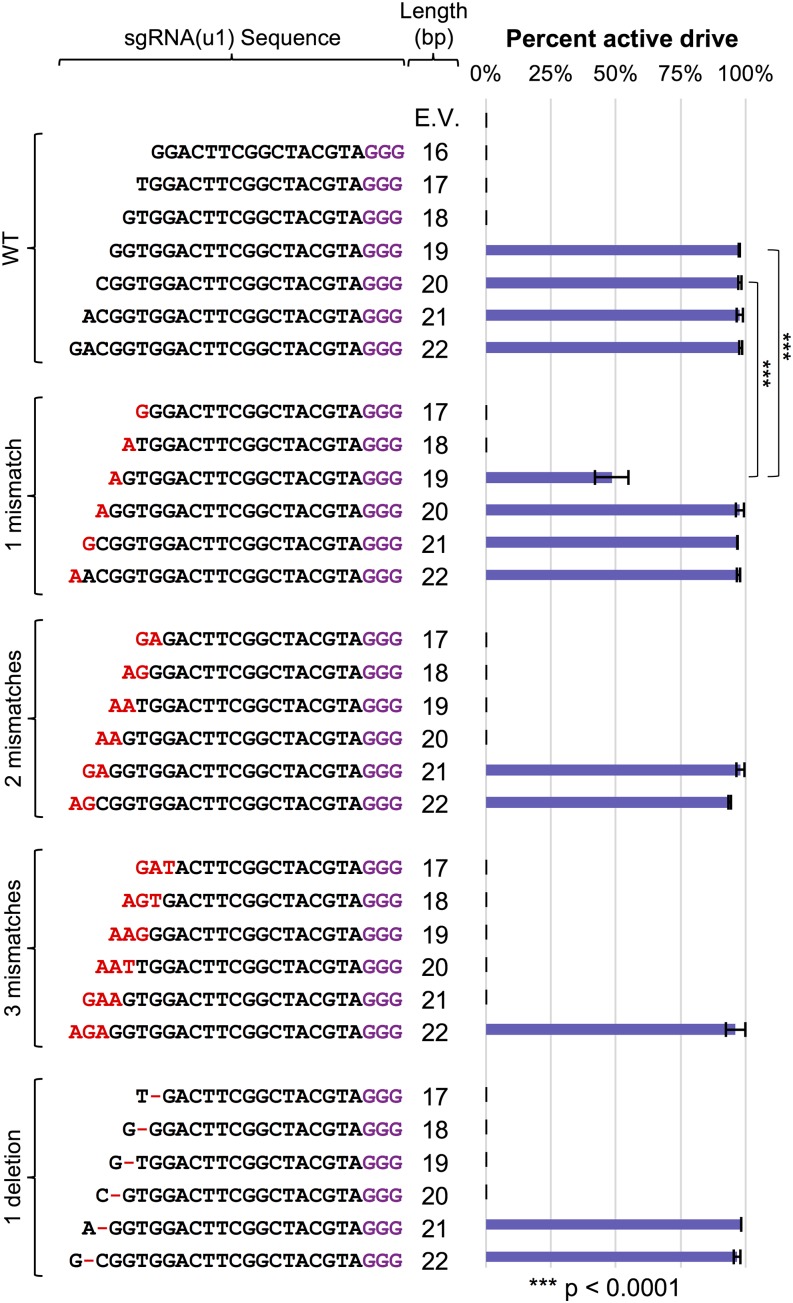
Varying sgRNA identity to control gene drives. GFY-2383 yeast was transformed with the collection of sgRNA(u1) cassettes from Figure 2. Yeast were mated to the target strains (GFY-3206 and GFY-3207), diploids selected, and drives were activated as described in Figure 5. Diploids were induced in YP + Galactose for 24 hr prior to plating in triplicate. For sgRNA(u1) 20 bps (WT) and 19 bps (one mismatch), six independent trials were performed (also see Figure S3 in File S1). The percentage of yeast colonies with an active gene drive was quantified. The total number of dead colonies on SD-HIS plates compared with the corresponding colonies on SD-LEU plates represented the active gene drive percentage. Error, SD. The two comparisons highlighted were analyzed using an unpaired *t*-test.

We also examined the effects of altering the sgRNA length and mismatch (Figure 2) in the context of our gene drive system (Figure 6). We obtained nearly identical results to our haploid editing experiments; only guide lengths of 19–22 allowed for an active gene drive even when the galactose induction time was increased to 24 hr. However, the 19 bp guide length with one 5′ mismatch (G→A) did provide the only partially functioning (∼45% active) drive system. We recognize that this is reduced compared with the efficiency of editing in haploid cells (80–85% effective) yet there are major differences in the mode of repair between the two assays (NHEJ with the option to re-edit multiple rounds *vs.* homologous recombination in a diploid genome for action of a gene drive). Altering the 5′ base to either C or T resulted in a near total loss of drive activity (Figure S2 in File S1).

**Figure 7:**
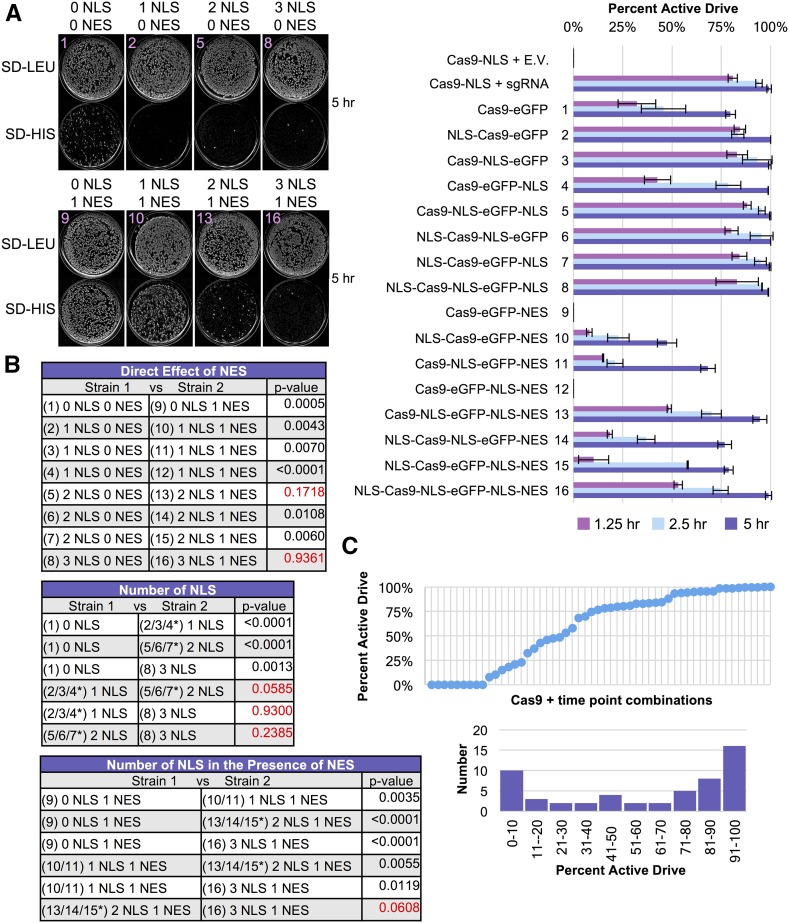
Modulation of gene drive activity by titration of Cas9 and nuclear shuttling. (A) Gene drives were generated based on the 16 plasmid-borne Cas9 constructs from Figure 3 and integrated at the *HIS3* locus. All gene drive strains (GFY-2751–GFY-2766) were transformed with the sgRNA(u1) 20 bp WT guide plasmid and mated to the two target strains (GFY-3206 and GFY-3207). Following diploid selection and preinduction in a raffinose/sucrose mixture, diploid yeast were cultured in YP + galactose for 1.25, 2.5, or 5.0 hr prior to plating. Representative plates (the Cas9-eGFP fusion number illustrated for clarity) for two groupings are illustrated at the 5 hr time point on SD-LEU and SD-HIS medium (left). The percentage of yeast with active gene drives (percentage of colonies dead on SD-HIS) was quantified in triplicate (right). Error, SD. (B) Two-way comparisons between strains from (A) were performed using an unpaired *t*-test. Red text, p-values > 0.05. Asterisk, the collective average of all three strains was used for comparisons. (C) The data from (A) was reordered from least to greatest percentage of active gene drive (top). The data from (A) is presented in a histogram with 10% binning categories (bottom).

We observed the same trend (lower gene drive activity when compared with haploid NHEJ-based editing) when we examined the Cas9-eGFP fusions containing various NLS/NES combinations (Figure 7). Given that our WT Cas9-based drive (Figure 5) was nearly 99% active after 4 hr postgalactose shift, we tested our 16 Cas9-eGFP variants at 1.25, 2.5, and 5.0 hr following induction (Figure 7A). Indeed, all constructs containing only added NLS signals (fusions 2–8) displayed nearly 99% active drive efficiencies after 5 hr of Cas9 expression; the Cas9-eGFP fusion with no added signal sequence was markedly lower at 75% (fusion 1). Addition of the C-terminal NES signal resulted in a dramatic reduction in nearly all drive activities (fusions 9–16). In fact, constructs containing only an NES or NLS-NES motif showed no activity at the 5 hr mark; however, this was not due to a lack of Cas9 expression (or nuclease activity) as these constructs were readily expressed, excluded from the nucleus, and were able to initiate editing in both haploid cells and gene drive diploids at a later time point (Figure S7 in File S1). The same general trend was observed for the competition between NLS and NES signals with additional NLS motifs providing higher drive activity (Figure 7B). These results highlight that titration of Cas9 protein expression with nucleocytoplasmic shuttling can provide a wide range of gene drive efficiencies from very low activity (0%) to WT levels (99%) (Figure 7C). Moreover, our findings demonstrate that nuclear exclusion (or limited residence time) may serve as a reasonable mechanism to fully inhibit or titrate drive activity (depending on the presence of other signals). Finally, given less time for Cas9 induction (<5 hr), there was still a modest increase in drive activity for constructs with two or three NLS sequences compared with only one nuclear signal, even in the absence of any NES (Figure 7).

**Figure 8:**
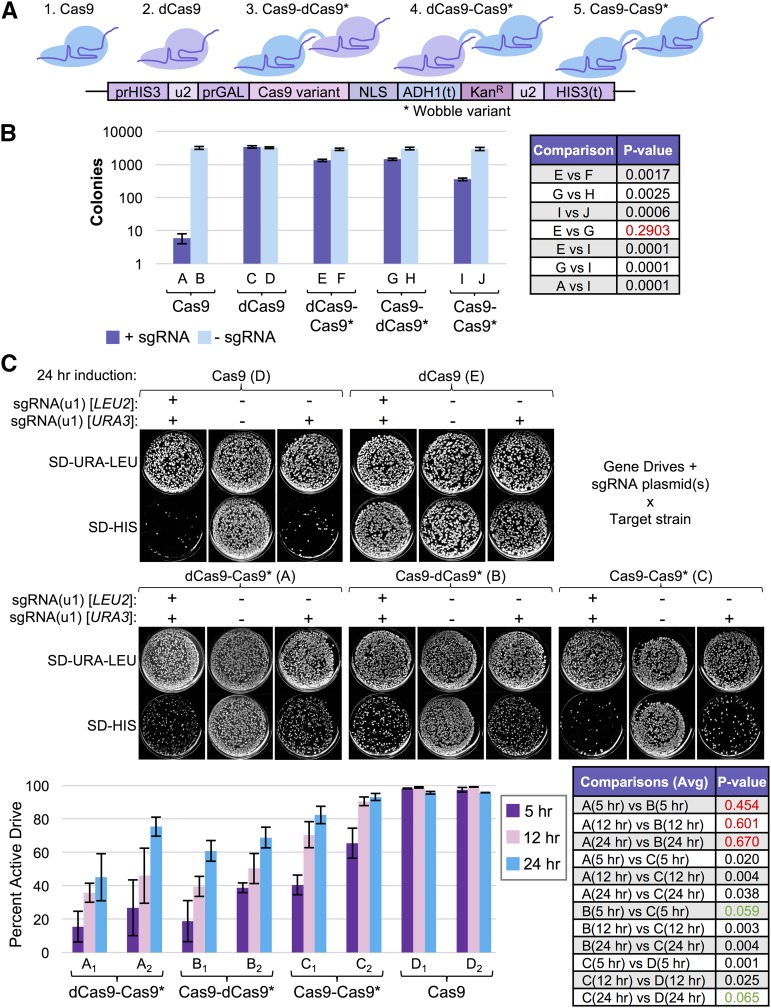
Novel fusions of enzymatically active and inactive Cas9 reduce gene drive activity. (A) Model of tandem Cas9 fusion design. A second Cas9 gene (asterisk) was synthesized *de novo* by altering >90% of the codons (primarily within the Wobble position). A 15-residue flexible linker was inserted between the two Cas9 copies. Dead Cas9 contains the mutations D10A and H840A. (B) GFY-2383, GFY-3250, GFY-3099, GFY-3100, and GFY-3336 yeast were transformed with equimolar amounts of either an empty vector (pRS425, duplicate), or a plasmid expressing the sgRNA(u2) 20 bp WT cassette (pGF-V809, triplicate), plated onto SD-LEU medium and incubated for 3 d. The total number of viable colonies were quantified (left). Error, SD. Two-strain comparisons were performed using an unpaired *t*-test (right). Red text, p-values > 0.05. (C) Yeast strains from (B) were each transformed with two plasmids (*URA3* and *LEU2* markers) resulting in four conditions: (i) sgRNA(u1)/sgRNA(u1), (ii) sgRNA(u1)/empty, (iii) empty/sgRNA(u1), and (iv) empty/empty. These included pRS425, pRS426, pGF-V1220, and pGF-V1625. Only the data for one of the sgRNA(u1)/empty combinations (pGF-V1625/pRS425) is presented. Strains were mated to the gene drive target strains (GFY-3206 and GFY-3207) and diploids were selected on SD-URA-LEU-HIS three consecutive rounds. Strains harboring either (i) two empty vectors or (ii) expressing a single copy of dCas9, were only mated to GFY-3206. Diploid yeast were preinduced overnight as previously described, and Cas9 expression was induced for 5, 12, or 24 hr in YPGal medium prior to dilution onto SD-URA-LEU plates. Finally, yeast were transferred to SD-URA-LEU and SD-HIS plates before imaging (top). The percentage of active gene drives (percentage of colonies dead on SD-HIS plates) was quantified (*bottom*). Error, SD. Comparisons between strains (all time points included) were performed using an unpaired *t*-test. p-values > 0.10 (red text), between 0.05 and 0.10 (green text), and <0.05 (black text). For individual time point comparisons, see Table S2 in File S1.

Finally, we tested whether enzymatically dead Cas9 (dCas9), which is still able to associate with the guide RNA and to target DNA, could serve as a direct competitor of active Cas9 (of the same species) when provided with an identical sgRNA (Figure 8A). Rather than construct two separate expression cassettes for active Cas9 and dCas9, we created three separate tandem fusions between *S. pyogenes* Cas9 and either a second active nuclease, or an enzymatically dead version for several reasons (while the protein products were identical, the coding sequences were altered to prevent inappropriate homologous recombination and potential loss of one copy) (Figure 8A). First, translational fusions to Cas9 (or dCas9) have been extensively used by many groups to add fluorescent proteins (Ma *et al.* 2015), chromatin-modifying enzymes (Morgan *et al.* 2017), transcriptional activators/repressors (Jensen *et al.* 2017), or other DNA-modifying enzymes (Kim *et al.* 2017). Second, a gene fusion between otherwise identical Cas9 enzymes would circumvent the need for exacting titration of transcript/translation for a direct one-to-one comparison. Third, incorporation of both Cas9 genes at the site of the gene drive (*HIS3* locus) would require the addition of unique promoter and terminator sequences flanking the secondary gene copy in a “separate” arrangement. Fourth, use of an additional yeast-specific (inducible) promoter sequence to drive expression of a second Cas9 variant would have limited utility to gene drive systems in other organisms. Thus, we have focused our initial efforts on tandem *S. pyogenes* Cas9 gene fusions, although we recognize that future iterations might consist of “competing” freely expressed Cas9 nucleases to titrate editing.

Transformation of these tandem Cas9-Cas9 fusions along with controls with the self-excising guide RNA (u2) demonstrated that both orientations (Cas9-dCas9 and dCas9-Cas9) resulted in a reduced level of editing in haploids compared with WT Cas9 expressed alone (Figure 8B). Interestingly, the tandem fusion of two nuclease-active Cas9 proteins (strain I) displayed a marked increase in editing compared with the dCas9-containing fusions yet was still significantly impeded when compared with free WT Cas9. We next examined these Cas9 variants in the context of our gene drive system. We hypothesized that one possible contributing factor to the overall reduction in editing with all Cas9 fusions could be the requirement of additional sgRNA fragments per Cas9 polypeptide: the fusion might serve as an sgRNA “sink” requiring double the level of RNA compared with free Cas9. Therefore, we tested three conditions at various time points using two identical sgRNA-expressing plasmids (marked with *URA3* or *LEU2*): (i) two empty vectors, (ii) two sgRNA(u1) plasmids, and (iii) one sgRNA/one empty plasmid (Figure 8C). Both combinations of the sgRNA/empty plasmid condition were tested and displayed nearly identical results (E. Roggenkamp, R. M. Giersch, M. N. Schrock, E. Turnquist, M. Halloran, and G. C. Finnigan, unpublished data). We found that the Cas9-dCas9 and dCas9-Cas9 fusions resulted in ∼45–70% drive activities (24 hr) (conditions A and B). Testing of the dual active Cas9-Cas9 fusion revealed a marked increase (24 hr, 80–90%) in overall drive activity, especially at earlier time points, yet still fell short of the optimal freely expressed WT Cas9 (24 hr, >95%) (condition C).

Interestingly, we observed only a modest contribution of having multiple guide RNA-expressing plasmids under all conditions tested, and the majority were not statistically significant (Table S2 in File S1) suggesting that, in our experimental setup (high-copy plasmid and *SNR52* promoter), there is likely near-sufficient guide RNA present. Next, there was no difference between the placement of the dCas9 variant on the N- or C-terminus of active Cas9 (both are separated by a 15-residue flexible linker). Also, use of the Cas9-Cas9 variant, identical save for two mutational substitutions, illustrated that there is a direct contribution of the second active nuclease within this arrangement and there is likely direct competition of the dCas9 variant within the mixed fusions.

**Figure 9:**
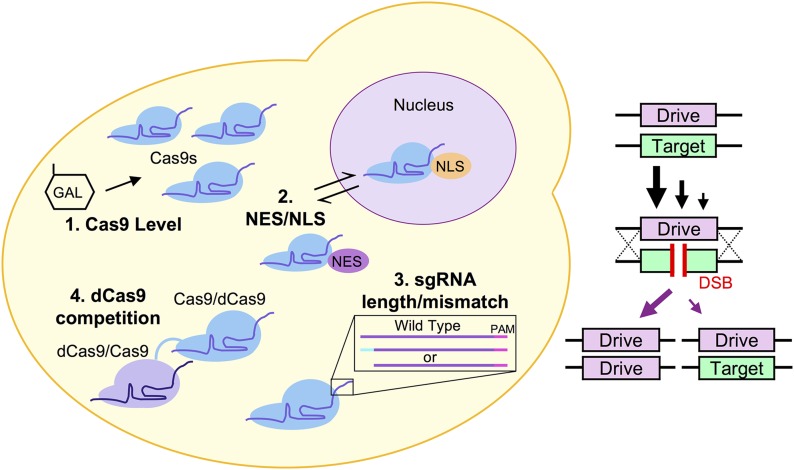
Model of four independent mechanisms for titration of Cas9-based gene drive activity. (1) Expression level of Cas9 protein from an inducible promoter can alter gene drive effectiveness. (2) The nucleocytoplasmic shuttling of Cas9, with varying NLS and NES signal combinations, provides a mechanism for achieving a wide range of gene drive activities ranging from 0 to 99%. (3) Altering the sgRNA length (19 bp) and level of mismatch (one mutation at the 5′ end), can reduce drive activity by ∼50%. This may be specific to the different substitutions. (4) A dual Cas9 fusion between active and dead Cas9 (in either orientation) or a tandem fusion of two active Cas9 proteins can also partially reduce drive activity. All four mechanisms can be combined and the effects on drive effectiveness compounded (left). Across a population, each of these mechanisms may result in a proportion of individuals that achieve proper activity and copying of the gene drive system; other individuals will be unable to propagate the drive in a super-Mendelian fashion (right). Together, these systems may be used to titrate a specific success (propagation) rate for a CRISPR gene drive within a population.

Finally, the decrease in drive activity (and haploid editing) of the Cas9-Cas9 fusion compared with free WT Cas9 may result from slowed nuclear import, as the expected molecular weight of the tandem fusions is expected to be nearly 320 kDa. However, passive diffusion of chimeric model proteins was found to exceed the 60 kDa limit in a previous study, suggesting that, perhaps, the nuclear pore complex can accommodate much larger proteins, especially those constructed from protein fusions, rather than natively assembled masses (Wang and Brattain 2007). Moreover, upper estimates of a eukaryotic nuclear pore complex cargo size included a diameter of up to 39 nm (using cargo-receptor-gold particle complexes) (Pante and Kann 2002), whereas the diameter of a single Cas9 protein is well below 15 nm (Jinek *et al.* 2014). In support of this model of slowed nuclear import, the earliest time point (5 hr) for this fusion displayed a dramatic difference with WT Cas9, yet given additional induction time (12 or 24 hr), the Cas9-Cas9 variant was able to achieve a significant level of editing and drive activity. Collectively, our results present four molecular aspects of CRISPR-based gene drives that have the potential to modulate—in a programmable, predictable fashion—the activity and effectiveness of a given gene drive system (Figure 9).

## Discussion

### Rationale for a “programmable” gene drive

The discovery and rapid expansion of CRISPR-based gene editing across all fields of molecular biology has led to many unique applications of this biotechnology. These range from “traditional” editing (deletion, modification, or replacement of genomic loci) (DiCarlo *et al.* 2013; Jinek *et al.* 2013), to genome-wide screens (Koike-Yusa *et al.* 2014; Wang *et al.* 2014), to repurposing of dCas9 to modulate gene transcription (Gilbert *et al.* 2013; Gao *et al.* 2016; Jensen *et al.* 2017), to imaging of chromosome dynamics in real-time (Ma *et al.* 2015, 2016b). Arguably one of the most powerful (and ethically concerning) uses of CRISPR-Cas9 is within the nuclease-based gene drive. This system has been recognized as means of biological control that could combat insect pests, invasive species and, most notably, insect-borne pathogens such as malaria. Indeed, early studies in the laboratory have demonstrated the unnatural power of a gene drive system to impose strong selection and over a 95% reduction in a population in only a few generations (Gantz *et al.* 2015; Hammond *et al.* 2016). Clearly, use of Cas9 in this genetic arrangement has the potential to impose a new level of control over native populations never seen before. However, early efforts have recently recognized several new challenges facing the development and application of gene drive–containing systems in the wild. First, given the incredibly strong selective pressure on a population, insect species have been shown to evolve resistance to the gene drive (Beaghton *et al.* 2017; Champer *et al.* 2017; Hammond *et al.* 2017; Noble *et al.* 2017). Second, wild populations may naturally have a diverse set of polymorphisms within a given gene target (and thus provide a native source to evade a gene drive) (Drury *et al.* 2017). Third, there is ongoing competition between HDR-based propagation of the drive and repair of the DSB via NHEJ (Prowse *et al.* 2017). Fourth, there are major ethical and scientific concerns regarding the implementation and use of a biological agent capable of (potentially) unrestricted population control on a large scale (Abbasi 2016; Reid and O’Brochta 2016; Adelman *et al.* 2017; Courtier-Orgogozo *et al.* 2017a,b; Ritchie and Johnson 2017; Wolt 2017).

We remain optimistic that the technical challenges facing gene drive success will be resolved given additional and ongoing laboratory study; indeed, the field is incredibly new and only a few labs have begun examining various drive designs. Moreover, there are already proposed solutions to increase the effectiveness of drives within wild populations including (i) multiple gene drives (and multiple targets), (ii) numerous sgRNAs (four or more to circumvent evolved resistance) (Marshall *et al.* 2017; Prowse *et al.* 2017), (iii) targeting of highly conserved/invariant (within a given species/subspecies) DNA sequences within a gene target to avoid polymorphisms (Burt 2003; Hammond *et al.* 2016), (iv) efforts to study and (eventually) disrupt the host-pathogen pairing rather than killing of the entire (host) insect (Gantz *et al.* 2015; Rénia and Goh 2016), and (v) other proposed drive arrangements including “anti-drive” drives (DiCarlo *et al.* 2015; Noble *et al.* 2017). Therefore, our goal was to investigate one major aspect of gene drives that has never been studied or considered to date: a methodology to program a desired drive effectiveness that is fully self-contained and requires no external regulatory mechanism.

We envision numerous reasons why a suboptimal drive arrangement may be desirable in practice. First, lowering the rate by which a gene drive can propagate through a given population would require more time to achieve full penetrance (*e.g.*, 10% effective drive *vs.* 99%). Future work will be required to carefully map the relationship between drive activity, organism generation time, and penetrance within a population; indeed, recent studies have begun using *in silico* modeling to examine the dynamics of gene drives within populations (Godfray *et al.* 2017; Noble *et al.* 2017; Unckless *et al.* 2017). A titratable drive system might be useful in the initial stages of field testing or studying optimization of a drive system in a native or controlled population: increasing the number of generations required for full conversion would allow for (i) control over the length of time required for full penetrance which may be of use for organisms with a very rapid (or too rapid) generation time and (ii) the option to counter, halt, or release a fail-safe drive to reverse, inhibit, or destroy the primary drive should the need arise. Population suppression (rather than elimination) is still a useful goal that might benefit from a tunable drive system (Godfray *et al.* 2017).

Second, the use of gene drives does not necessarily have to be restricted to application within wild populations. Indeed, several studies have demonstrated that gene drives represent powerful genetic screening tools that can be used within basic laboratory research (Roggenkamp *et al.* 2017; Shapiro *et al.* 2018). A tunable gene drive could be useful in studying population dynamics, evolved resistance, or in the generation of a heterogeneous population of edited cells/cell types for use in high throughput screening. Third, nearly every form of biological control (*e.g.*, especially that used in agriculture such as natural predators, chemical agents, or physical barriers/traps) of pests or pathogens includes the ability to titrate the proposed solution to a level that is safe (to surrounding plant and animal life and to humans), cost-effective, and appropriate given the nature of the problem at hand. In its current state, deployment of an active gene drive would have two levels: 0% (not active) or nearly 99% (fully active) rates. Fourth, components that can partially reduce/slow drive activity may be able to selectively, or in combination, impose maximum reduction in drive activity should the need arise and serve as a controllable and inducible (temporary or permeant) off switch. Therefore, we have performed an investigation into invariant components of all gene drive systems regardless of the proposed organism of use: Cas9 and the sgRNA.

### Molecular mechanisms to tune drive activity in yeast

Use of *S. cerevisiae* as a model system to study gene drives comes with the numerous benefits of this popular and genetically tractable model organism. As a model eukaryote, many have begun to use yeast to demonstrate novel applications and uses of Cas9, including synthetic genome construction (Tsarmpopoulos *et al.* 2016), chromosome splitting (Sasano *et al.* 2016), dCas9 transcriptional modulation (Jensen *et al.* 2017), reengineering of biosynthetic pathways (Ryan and Cate 2014; Kang *et al.* 2016) and, recently, gene drives (DiCarlo *et al.* 2015; Roggenkamp *et al.* 2017). We have developed a system for examination of Cas9 editing both in a haploid genome and in the diploid state (for gene drive activity). This model system provides several important benefits to the study of gene drives: the risk of unintended (or malicious) drive release is minimized because laboratory yeast do not sporulate well, are not airborne and, as we have documented here, can be programmed with multiple genetic safeguards that render any escaped drive inviable. These simple, yet powerful genetic additions (our artificial “unique” target sequences and self-excising sequences flanking Cas9 itself) ensure no native yeast strain or population would ever be inappropriately targeted by the gene drive. Moreover, as the Church laboratory demonstrated (DiCarlo *et al.* 2015), and we document as well, separation of the sgRNA (on a plasmid) serves as a potent natural failsafe for studying of gene drives in yeast. Finally, the rapid lifecycle, molecular tools available to the yeast community, and high-throughput infrastructure (Duina *et al.* 2014) allow for a true exploration and investigation of hundreds, if not thousands, of gene drive arrangements to be tested. Given the technical challenges to constructing only one or two viable drive systems in insects (Champer *et al.* 2017), it would be extremely difficult to explore more than a single variable given the challenging and time-consuming *in vivo* systems in arthropods or higher eukaryotes.

Here, we explored four conserved mechanisms of the CRISPR system and demonstrate that all four are independent means to titrate gene drive activity within our yeast system. We envision further development of each of these modes of control to varying degrees. While an inducible promoter system driving Cas9 transcription may not be a practical solution to study/test in an insect model, this could still be useful in promoter choice, and possibly complex modulation of the Cas9 promoter itself. For instance, we envision that multiple layers of transcriptional regulation could be assembled onto the “primary” Cas9 nuclease used to initiate the gene drive by a secondary copy of dCas9 fused to either an activator or repressor to modulate expression. Moreover, numerous evolved variants of Cas9 are now paired with either an inducible promoter or external stimuli including small molecules, temperature, and even light (Dow *et al.* 2015; Polstein and Gersbach 2015; Zetsche *et al.* 2015; Cao *et al.* 2016; Liu *et al.* 2016; Nuñez *et al.* 2016; Richter *et al.* 2016).

Our sampling of sgRNA lengths and identities was by no means comprehensive. A plethora of studies have extensively tested many variables surrounding sgRNA design: single or two-part guides, length, mismatch, deletions, stem-loop identities, and chemical modifications or fusions (Briner *et al.* 2014; Lin *et al.* 2014; Anderson *et al.* 2015; Dang *et al.* 2015; Farboud and Meyer 2015; Moreno-Mateos *et al.* 2015; Briner and Barrangou 2016; Doench *et al.* 2016; Fu *et al.* 2016; Smith *et al.* 2016). Based on our limited set, and given further study, we are hopeful that there is a viable population of guide RNA options that might produce a titratable level of editing (like our 19 bp guide with a single 5′ G→A mismatch) in the context of a drive system. Importantly, other groups have also reported varying levels of editing given distinct mismatches within the two most 5′ bases of the crRNA sequence (Anderson *et al.* 2015; Zheng *et al.* 2017) as we have demonstrated in this study. This might be explained by varied expression, stability, and/or Cas9 loading of the RNA. The commonly used U6 promoter requires a 5′ G base pair for expression (Cong *et al.* 2013; Fu *et al.* 2013), although crRNA transcript levels might also be affected by other positions within the guide, including the seed region (Wu *et al.* 2014).

The primary finding of our study involves titration of Cas9 nuclear occupancy through the active nucleocytoplasmic shuttling achieved by presentation of multiple NLS or NES signal(s). Nuclear Cas9/sgRNA complex residence time has been shown to limit editing efficiency (Ma *et al.* 2016a). Given that the mechanisms for nuclear transport of proteins are conserved (Lange *et al.* 2007; Freitas and Cunha 2009; Bauer *et al.* 2015) and that canonical signal sequences including, but not restricted to, the SV40 NLS (Kalderon *et al.* 1984) are used across model systems, we envision this as a viable and rich option for modulation of editing in gene drives. The increased contribution to promoting Cas9 editing has already been demonstrated in other cell types and current Cas9 systems use two or three NLS sequences to maximize editing (Ménoret *et al.* 2015; Torres-Ruiz *et al.* 2017). In our designed system, using the potent *GAL1/10* promoter, a single SV40 NLS appeared sufficient to direct trafficking of Cas9 in either haploid cells or diploid gene drive setups. However, our work demonstrates that, like other cell systems that often require more than one nuclear signal, multiple NLS sequences provide a more robust import signal when challenged with either a suboptimal degree of Cas9 expression and/or opposing nuclease export signal. Of note, our genetically encoded Cas9 system uses one of the highest expressed promoters in yeast (Flick and Johnston 1990), and other CRISPR editing systems utilize a variety of means of Cas9 delivery, including chromosomally encoded Cas9, plasmid-expressed, or microinjected purified Cas9/sgRNA ribonucleoprotein. Furthermore, different cell types have been shown to display highly variable Cas9 localizations regardless of the presence of one or two NLS sequences (Torres-Ruiz *et al.* 2017), making a direct comparison across systems challenging. Finally, the placement of NLS signal sequences (distance from the nuclease coding sequence) may also be a factor in accessibility of nuclear import machinery (Shen *et al.* 2013) as well as context to the fused protein of interest (Nelson and Silver 1989). However, as a general strategy, our study and others (Cong *et al.* 2013) have concluded that the addition of more than one NLS sequence can serve to increase nuclear localization and editing, and may buffer against other factors that could interfere with optimal import. The identification and characterization of endogenous NLS signal sequences (Weninger *et al.* 2015) specific to the organism of interest would also provide an additional suite of options for either optimized or titratable nuclear import and subsequent Cas9 editing. Using the dynamic nuclear import/export of Cas9, we have demonstrated that both the level of Cas9 and its nuclear occupancy can modulate drive activity over a wide range of efficiencies. In fact, one possible mechanism for shutoff (or reduction) of Cas9 editing could be induced attachment or recruitment of a NES-containing peptide or protein and subsequent nuclear exclusion. This could even be coupled with the newly discovered “anti-CRISPR” family of short peptides that serve to directly bind to and inhibit Cas9 nuclease activity (Pawluk *et al.* 2016; Dong *et al.* 2017; Rauch *et al.* 2017; Shin *et al.* 2017; Yang and Patel 2017).

Finally, we have piloted the use of a set of Cas9-Cas9 fusions to illustrate that (i) dCas9 can compete with native nuclease-active Cas9 and (ii) the large size of a tandem active fusion can partially impede editing. We recognize that alternative Cas9 orthologs (Esvelt *et al.* 2013; Karvelis *et al.* 2017) would also provide a unique mode of titration between one active and one dead nuclease (or other combinations therein) competing for identical or nearby target sequences. Our system was developed because (i) only a single sgRNA cassette was required, (ii) only a single gene module was needed to express the dual Cas9 fusion (rather than two separate or identical promoters), (iii) a flexible linker provided both the N- and C-terminal nucleases ample spacing, and (iv) addition of coding sequence as part of a gene fusion could be directly applicable to gene drive systems in other organisms. We are excited about the potential that unique Cas9 fusions may serve within the context of gene drives given the rapid explosion of new variants that currently exist. Indeed, dCas9 has provided the expansion of an entirely new field of fusing other enzymes of interest from DNA modifying enzymes, to transcriptional regulators, to “base editors,” to fluorescent proteins, to other nuclease enzymes (Guilinger *et al.* 2014; Bolukbasi *et al.* 2015; Ma *et al.* 2015; Thakore *et al.* 2015; Chaikind *et al.* 2016; Nuñez *et al.* 2016; Clements *et al.* 2017; Kim *et al.* 2017; Lin *et al.* 2017; Zong *et al.* 2017). Similarly, placement of different arrangements of Cas9 fusions (or expressed as separate proteins, or a cleavable fusion), different linker lengths or restrictions between fused proteins, or the presence of other nonnuclease modifying enzymes or tags within the drive system could aid to optimize, inhibit, or, as we have demonstrated, titrate the level of overall drive activity.

Given the technical, societal, and ethical challenges facing application of gene drives in the wild, additional study in controlled laboratory settings is critical. Our yeast drive system represents a safe, contained, and rapid testing platform to explore the numerous new Cas9 variants, sgRNA arrangements, and the subcellular trafficking of the Cas9/sgRNA complex to identify new means for future control, regulation, or inhibition in fungi, plant, or metazoan hosts and possible application in wild populations.

## Supplementary Material

Supplemental material is available online at www.g3journal.org/lookup/suppl/doi:10.1534/g3.117.300557/-/DC1.
