# Supplementary material for: Tuning CRISPR-Cas9 Gene Drives in Saccharomyces cerevisiae
Source: G3 (Bethesda). 2018 Jan 18;8(3):999–1018. doi: 10.1534/g3.117.300557 (PMC5844318; doi:10.1534/g3.117.300557)
Supplement: Supplementary file 1 [file 999FileS1.docx]

**SUPPORTING INFORMATION**

For

**Tuning CRISPR-Cas9 gene drives in *Saccharomyces cerevisiae***

Emily Roggenkamp,^1†^ Rachael M. Giersch,^2†^ Madison N. Schrock,^1,2†^ Emily Turnquist,^1^ Megan Halloran,^1^ and Gregory C. Finnigan^1*^

^1^Department of Biochemistry and Molecular Biophysics, 141 Chalmers Hall, Kansas State University, Manhattan, KS 66506 USA

^2^Department of Biology, 116 Ackert Hall, Kansas State University, Manhattan, KS 66506 USA

^†^Authors contributed equally

*Correspondence to: Gregory C. Finnigan, Phone: (785) 532-6939; FAX; (785) 532-7278; E-mail: [gfinnigan@ksu.edu](mailto:gfinnigan@ksu.edu)

**Figure S1.** Editing of haploid yeast with a greatly increased Cas9 induction time.

(A) GFY-2588 yeast containing pGF-IVL1342 (inducible Cas9) were transformed with sgRNA(u1) plasmid (pGF-V1220) and incubated overnight (16 hr) in YPGal as previous described (Fig. 1). For 6 independent trials, yeast were plated onto SD-URA-LEU media for 4 days. For 29 additional independent experiments, yeast were plated onto SGAL-URA-LEU. The total number of colonies across all treatments was quantified (*top*). Selection on galactose-containing plates (>4 days) resulted in nearly a 10-fold reduction of viable colonies following Cas9-based editing. All surviving colonies (14 for dextrose treatment, 1-14, and 8 for galactose treatment, 15-22) were selected a second time (identical plate condition) as single clonal isolates and tested for G418-resistance (*bottom*). (B) Chromosomal DNA was prepared, PCR amplified at the *HIS3* locus, and assayed by Sanger DNA sequencing. For dextrose-treated isolates (14), a variety of sequences were obtained including the original unmodified target cassette and dual (u1) sites, or various insertions/deletions. However, for galactose-treated isolates (8), only the unmodified target cassette (with Kan^R^) was obtained.

**Figure S2.** Editing with a modified 19-base pair sgRNA.

(A) Haploid editing with a modified 19-bp sgRNA. GFY-2588 haploid yeast harboring pGF-IVL1342 (inducible Cas9) were grown overnight to saturation in a raffinose/sucrose mixture and back-diluted into YPGal medium for 4.5 hrs in triplicate. Cell were harvested, washed, and transformed with 1000 ng of sgRNA(u1) plasmid harboring a mutation at the 5’ end (19 bp guide sequence; pGF-V1225, pGF-V1797, or pGF-V1799, triplicate) or control plasmids (pGF-V1220, 20 bp WT sgRNA or pRS425, empty vector, duplicate). Following recovery in fresh YPGal overnight, cells were plated onto SD-LEU-URA and incubated for 3 days prior to imaging and quantification. A sampling of colonies was tested on media containing G418 to assess the percentage of cells that had excised the Kan^R^ marker at the *HIS3* locus. (B) Gene drive function with a modified 19-bp sgRNA. GFY-2383 yeast were transformed with high-copy sgRNA(u1)-expressing plasmids with a 19 base pair guide sequence containing a single substitution at the 5’ end as in (A). Yeast were mated to the gene drive target strains of the opposite mating type (GFY-3206 and GFY-3207) in quadruplicate and diploids were selected on SD-LEU-HIS media (three consecutive selection steps). Strains were pre-induced overnight (raffinose/sucrose mixture) and grown in rich medium containing galactose for 5 hr prior to plating onto SD-LEU plates (500-1000 cells per plate). Colonies were replica-plated to both SD-LEU and SD-HIS after two days of growth and grown for an additional 24 hr before imaging. Representative plates are shown. The percentage of colonies with active drives is illustrated for sample plates (red text).

**Figure S3.** Examining gene editing with GFP-tagged and catalytically dead Cas9 in haploid yeast.

Yeast (GFY-2353) containing the Hyg^R^ cassette flanked by the artificial (u1) sites were transformed with plasmids containing Cas9-NLS (pGF-IVL1116), dCas9-NLS (pGF-IVL1180), Cas9-eGFP-NLS (pGF-IVL1119), or dCas9-eGFP-NLS (pGF-IVL1183), cultured overnight in pre-induction media (raffinose/sucrose), back-diluted into YP + galactose for 4.5 hr, and transformed with either the sgRNA(u1) plasmid (pGF-V1220) or an empty pRS425 vector, recovered overnight in galactose, and plated to SD-URA-LEU medium. The total number of colonies was quantified after 3 days at 30°C. Error, SD. Addition of the D10A and H840A mutations to Cas9 (catalytically dead Cas9) prevented any editing. Fusion of eGFP between Cas9 and a C-terminal NLS did not affect the ability of Cas9 to edit the yeast genome.

**Figure S4.** Correlation between phenotype (loss of marker) following gene drive action and genotype (*HIS3* locus).

(A) GFY-2583 yeast harboring the high-copy sgRNA(u1) plasmid, pGF-V1220, were mated with the gene drive target strains, GFY-3206 and GFY-3207, and diploids were selected on SD-LEU-HIS medium (identical protocol as Fig. 5). Following an overnight culture in pre-induction medium (raffinose/sucrose), strains were cultured in rich media containing galactose for 0, 1, 2, 4, or 8 hr before being plated onto SD-LEU (roughly 500-1000 cells per plate). Colonies were replica-plated to SD-LEU and SD-HIS medium and incubated for 24 hrs prior to imaging (*top left*). (B) Individual clonal isolates were obtained from the SD-LEU condition for each time point. A summary of the total number of isolates tested (including from Fig. 5) is provided. (C) Clonal isolates were tested for (i) ploidy status, (ii) G418 resistance, and (iii) growth on SD-HIS. Chromosomal DNA was isolated and subjected to four independent diagnostic PCRs to assess the status of the *HIS3* locus following action of the gene drive at each condition. The Cas9 gene drive was marked with the Kan^R^ cassette (G418 resistance) whereas the target locus included the *S. pombe HIS5* gene. PCRs A-D are identical to Fig. 5 (A and B assay the presence of the Cas9 gene drive whereas C and D are specific to the target locus). Including the data presented in Fig. 5, a total of 10 HIS+ diploids (all from the 0 hr time point) and 39 HIS- diploids (across multiple time points), each confirmed by four PCRs to the *HIS3* locus, demonstrate action of the gene drive and correlation between phenotype and genotype.

**Figure S5.** Spontaneous loss of high-copy sgRNA plasmid.

Yeast (GFY-2383) were transformed with the sgRNA(u1) expression cassette on the high-copy pRS425 vector (pGF-IVL1220), mated to the gene drive “target” strain (GFY-3206), diploids selected (SD-LEU-HIS medium), and Cas9 was pre-induced (raffinose/sucrose) overnight, and (with no activation of Cas9), directly plated to SD-LEU medium (to maintain the plasmid), and incubated for 3 days. A random sampling of colonies from the SD-LEU plate was separated into single colonies on rich medium (YP + Dextrose) and grown for 3 additional days. From this YPD plate, a random sampling of individual clonal isolates was chosen (n = 60-80 colonies), moved to a fresh YPD plate (*left*), incubated for 24 hr, replica-plated to SD-LEU (*right*), and grown for 24 hr prior to imaging. The number of clonal isolates sensitive on the SD-LEU condition was quantified (*far right*) in duplicate. Error, SD. From the remaining yeast on the YPD plate, a random sampling (full streak across the plate) was taken and propagated to a fresh YPD plate to isolate a new round of single colonies. The process was repeated 3 times on successive rounds of YPD plates. By the third round of colony isolation, nearly 80% of isolates had lost the pRS425-based plasmid (in less than 2 weeks) in the absence of any selective pressure or counter-selection.

** Figure S6.** Safeguarding gene drives in yeast—self-excision of Cas9 in haploids and diploids.

Our Cas9 gene drives all contain a pre-programmed genetic mechanism to active self-excision in either haploid or diploid cells (see Fig. 5A). We have purposefully inserted two artificial sites (u2) flanking the entire Cas9 drive cassette in all our gene drive configurations. These (u2) sites also contain a maximum mismatch to the *S. cerevisiae* genome (Finnigan and Thorner 2016) and are distinct from the (u1) sites built into the target strains (these have been omitted from the diagrams for clarity). (A) We demonstrate five independent means to activate self-excision of Cas9 from the genome using the (u2) sites and a plasmid expressing the sgRNA(u2) guide sequence. **WT**, the traditional mode of action for all gene drives. **Scenario 1**, haploid yeast containing Cas9 in the gene drive strain (GFY-2383) were mated to the target strain of the opposite mating type (GFY-3206) harboring the sgRNA(u2) plasmid (pGF-V809) with a *LEU2* marker, and diploids were selected twice. Following activation of the drive (pre-induction overnight followed by 24 hr in galactose medium), approximately 2,000 to 4,000 cells were plated to SD-LEU, grown for four days at 30°C, and transferred to fresh SD-LEU and G418 medium in triplicate (B, *lower* *middle*). Total colonies were quantified (B, *bottom, right*). Error, SD. Action of Cas9 on the flanking (u2) sites caused self-excision of the gene drive expression cassette and removal of the Kan^R^ marker by replacement with the target strain cassette (His+). We achieved >99.9% removal of the gene drive. **Scenario 2**, an identical procedure was performed with the same gene drive strain as in Scenario 1, but rather than the *MAT****⍺*** target strain, WT BY4742 yeast were used harboring the same *LEU2-*marked sgRNA(u2). The *HIS3* locus, following self-excision of the drive, would be homozygous diploid for the *his3∆1* allele (His-). **Scenario 3**, the same protocol as Scenario 2 was performed, but rather than a pRS425 vector containing the sgRNA(u2) cassette, a *HIS3*-containing pRS423 plasmid (pGF-V798) was included in the BY4742 strain for delivery to the Cas9 gene drive diploid. This plasmid includes 317 bp of 5’ UTR and 201 bp of 3’ UTR flanking genomic sequence to the *HIS3* locus and therefore serves as a source of donor DNA for repair of the DSB. Homology directed repair could occur through HR off the plasmid *HIS3* sequence, or from action of the *his3∆1* allele present on the homologous chromosome—in either scenario, the Cas9 drive is excised and removed (note, all cells would remain His+ due to the presence of the pRS423 plasmid). **Scenario 4**, the haploid gene drive strain (GFY-2383) was directly transformed with the sgRNA(u2) on pRS423 (pGF-V798), induced for expression, and plated onto SD-HIS medium prior to assaying on G418 plates. Self-excision of Cas9 was coupled with repair of the DSB by the provided *HIS3* donor DNA on the pRS423 plasmid (cells His+). **Scenario 5**, the haploid gene drive strain was transformed with the *LEU2*-based sgRNA(u2) plasmid and editing was initiated as previously described. Yeast were plated onto SD-LEU medium and action of Cas9 excises itself out of the genome; there is no donor DNA present to repair the DSB and surviving colonies rely on NHEJ. We estimate that 96-98% of edited yeast (plates not shown) were inviable following action of Cas9 (B, *bottom*, *right*). Together, our system provides several options to approach destruction and removal of the gene drive itself in either a haploid state (by direct addition of the guide RNA), or by introduction of a “suiciding” strain of the opposite mating designed to deliver the self-excising guide RNA. Moreover, our gene drive suicide system utilizes the artificial (u2) sites, and does not require targeting of any native yeast genomic sequence nor any other aspect native to the gene drive or the Cas9 gene itself. Our system is distinct from a previous method describing use of a second gene drive to destroy an initial drive-containing strain (DiCarlo *et al.* 2015) because (i) *any* yeast strain of the opposite mating type can serve as the delivery mechanism of the sgRNA(u2) plasmid, (ii) no additional Cas9 drive is required—the original drive “self-excises” itself from the genome, (iii) our safety mechanism utilizes non-native DNA targets (u2) and would not present any risk of off-target or inappropriate editing, and (iv) our system *could* include a secondary Cas9 drive (say, under a distinct promoter sequence) to selectively target, edit, and destroy any intended (original) drive set up. These data demonstrate a powerful mechanism for destruction of our programmed gene drives in either the haploid or diploid state.

**Figure S7.** Partial editing by the Cas9-eGFP fusions containing a C-terminal NES signal.

(A) Yeast (GFY-3101 and GFY-2758) were assayed for self-excision by using a sgRNA(u2)-expression plasmid (pGF-V809) that targets two artificial (u2) sites (Finnigan and Thorner 2016) flanking all gene drive cassettes at the *HIS3* locus. Strains were pre-induced in a synthetic raffinose/sucrose mixture, back-diluted into YP + galactose, and transformed with equimolar amounts of either empty pRS425 vector or sgRNA(u2) vector, recovered overnight in galactose, and plated to SD-LEU medium for 3 days at 30°C. Colonies were quantified in triplicate. Error, SD. An unpaired t-test was used to compare the final colony counts. While the decrease in the total number of colonies was modest, these data illustrate there is (some) action of Cas9 for both the NES or NLS-NES tagged Cas9-eGFP fusions. (B) Haploid strains from (A) as well as a WT Cas9 control strain (GFY-2383) all containing the sgRNA(u1) plasmid, were mated to the gene drive “target” strains (GFY-3206 and GFY-3207), diploids selected, Cas9 expression was pre-induced (raffinose/sucrose) overnight, and yeast were cultured in YP + galactose for 24 hr prior to plating onto SD-LEU medium. After 3 days, clonal isolates (randomly selected) were re-tested on SD-LEU plates and assayed for sensitivity on SD-HIS (between 50-75 colonies were tested for each strain) in quadruplicate. Error, SD. These data illustrate that both Cas9-eGFP-NES and Cas9-eGFP-NLS-NES display some gene drive activity, but greatly reduced compared to the action of WT Cas9-NLS. Moreover, our assessment of colonies from SD-LEU onto SD-HIS is likely an overestimate of drive activity since there is the possibility of clonal siblings to be accidently assayed. Regardless, these data present that there is gene drive action for the two NES-containing Cas9 constructs, albeit much lower, and requiring a 24 hr induction. (C) Strains (GFY-3270 and GFY-3271) were constructed based on those used in (A) that contain an integrated *NUP188*-mCherry to mark the nuclear periphery. Yeast were pre-induced overnight (raffinose/sucrose), back-diluted into YP + galactose for 4.5 hr, and imaged by fluorescence microscopy. White dotted lines, cell periphery. Scale bar, 3 μm. A faint circular haze seen in the mCherry channel represents the yeast vacuole (co-localized with the circular indentation in the DIC image). Representative images are shown; all exposure times are identical to those from Fig. 3. Steady state levels of Cas9-eGFP are excluded from the nucleus in both strains.

Table S1. Oligonucleotides used for diagnostic PCR.

| **Name** | **Fig.** | **Description** | **DNA Sequence (5’ to 3’)** |
| --- | --- | --- | --- |
| F1 | 1 | pr*HIS3* +196 F | GGCCTCCTCTAGTACACTCTATATTTTTTTATGC |
| R1 | 1 | *HIS3*(t) -152 R | GCGCCTCGTTCAGAATGACACGTATAGAAT |
| F1 | 5 | pr*GAL1/10* +192 F | GGGGTAATTAATCAGCGAAGCGATGATTTTTG |
| R1 | 5 | SpCas9 Int +373 R | CATCAACGATGTTACCGAAGATTGGATGTC |
| F2 | 5 | SpCas9 Int +3653 F | CGGTAGAAAAAGAATGTTAGCTTCAGCTGG |
| R2 | 5 | Internal Kan^R^ R | GAACACTGCCAGCGCATCAACAATATTTTC |
| F3 | 5 | pr*HIS3* +196 F | GGCCTCCTCTAGTACACTCTATATTTTTTTATGC |
| R3 | 5 | *SHS1*(t) -192 R | GCCATATTTAAATTTATCCCTACAATTATTTGACACTGTTTG |
| F4 | 5 | Int pr*CCW12* F | CGTACAAGTATTTCTCAGGAGTAAAAAAACCGTTTG |
| R4 | 5 | Int *S. pombe* *HIS5* R1 | CTGCTTGAATGCAATACCAAGTGCAATAGCAG |

Table S2. Unpaired T-test comparisons between gene drive activities for Cas9 fusion variants.

| **Comparison** | **P-value** |
| --- | --- |
| A2-5 versus A1-5 | 0.490 |
| A2-12 versus A1-12 | 0.497 |
| A2-24 versus A1-24 | 0.107 |
|  |  |
| B2-5 versus B1-5 | 0.155 |
| B2-12 versus B1-12 | 0.296 |
| B2-24 versus B1-24 | 0.327 |
|  |  |
| C2-5 versus C1-5 | 0.081 |
| C2-12 versus C1-12 | 0.752 |
| C2-24 versus C1-24 | 0.114 |
|  |  |
| D2-5 versus D1-5 | 0.536 |
| D2-12 versus D1-12 | 0.323 |
| D2-24 versus D1-24 | 0.885 |
|  |  |
| A1-5 versus B1-5 | 0.790 |
| A1-12 versus B1-12 | 0.586 |
| A1-24 versus B1-24 | 0.285 |
|  |  |
| A2-5 versus B2-5 | 0.424 |
| A2-12 versus B2-12 | 0.777 |
| A2-24 versus B2-24 | 0.389 |
|  |  |
| A1-5 versus C1-5 | 0.084 |
| A1-12 versus C1-12 | 0.036 |
| A1-24 versus C1-24 | 0.073 |
|  |  |
| A2-5 versus C2-5 | 0.102 |
| A2-12 versus C2-12 | 0.063 |
| A2-24 versus C2-24 | 0.053 |
|  |  |
| B1-5 versus C1-5 | 0.154 |
| B1-12 versus C1-12 | 0.047 |
| B1-24 versus C1-24 | 0.065 |
|  |  |
| B2-5 versus C2-5 | 0.057 |
| B2-12 versus C2-12 | 0.026 |
| B2-24 versus C2-24 | 0.035 |
|  |  |
| C1-5 versus D1-5 | 0.005 |
| C1-12 versus D1-12 | 0.036 |
| C1-24 versus D1-24 | 0.071 |
|  |  |
| C2-5 versus D2-5 | 0.038 |
| C2-12 versus D2-12 | 0.043 |
| C2-24 versus D2-24 | 0.226 |

For p-values greater than 0.10, red text was used. For p-values between 0.050 and 0.100, green text was used. For p-values < 0.050, black text was used. Yeast strains (A-D) correspond to the nomenclature used in Fig. 8C (A, dCas9-Cas9 fusion; B, Cas9-dCas9 fusion; C, Cas9-Cas9 fusion, and D, freely expressed WT Cas9). The number designation (e.g. A1, A2) refers to the presence of either 1 or 2 identical sgRNA-expressing plasmids (e.g. A2 contains both pRS425-based and pRS426-based sgRNA(u1) cassettes). For consistency, strains with only 1 sgRNA plasmid also harbor the corresponding high-copy empty vector. The final number indicates the induction time in galactose for each gene drive experiment (5, 12, or 24 hr).

**Works Cited**

DiCarlo, J. E., A. Chavez, S. L. Dietz, K. M. Esvelt and G. M. Church, 2015 Safeguarding CRISPR-Cas9 gene drives in yeast. Nat Biotechnol 33**:** 1250-1255.

Finnigan, G. C., and J. Thorner, 2016 mCAL: A New Approach for Versatile Multiplex Action of Cas9 Using One sgRNA and Loci Flanked by a Programmed Target Sequence. G3 (Bethesda) 6**:** 2147-2156.
